# A UFMylation-COPII axis orchestrates lipid transport in intestinal enterocytes and regulates systemic lipid balance

**DOI:** 10.1016/j.molmet.2026.102397

**Published:** 2026-06-12

**Authors:** Yaqun Wang, Feng Zhou, Xin Xu, Guangyu Wu, Hong Xu, Honglin Li

**Affiliations:** 1 Department of Physiology, School of Basic Medical Sciences, Jiangxi Medical College, Nanchang University, Nanchang, Jiangxi, China; 2 Department of Biochemistry & Molecular Biology, Georgia Cancer Center, Medical College of Georgia, Augusta University, Augusta, GA, USA; 3 Department of Pharmacology and Toxicology, Medical College of Georgia, Augusta University, Augusta, GA, USA

**Keywords:** UFMylation, Enterocyte, Chylomicron, COPII vesicle

## Abstract

**Background:**

Intestinal lipid absorption and chylomicron secretion are essential for systemic lipid homeostasis, yet the regulatory mechanisms coordinating lipoprotein assembly and ER export remain poorly understood. UFMylation is a newly identified ubiquitin-like modification pathway that plays critical roles in endoplasmic reticulum (ER)-related cellular activities such as protein quality control, ER-associated degradation (ERAD) and ER-phagy. However, its role in intestinal lipid transport and systemic lipid homeostasis is completely unclear.

**Methods:**

To elucidate the role of UFMylation in intestinal lipid metabolism, we generated intestinal epithelial cell (IEC)-specific knockout mouse model of *Ufbp1*, a key component of the UFMylation pathway, and a double knockout model of *Ufbp1* and *IRE1α*, one of the three signaling branches of Unfolded Protein response (UPR). After observing lipid droplet accumulation in the intestinal tissue of *Ufbp1* and *IRE1α* double knockout mice, we further examined lipid metabolism in *Ufbp1* knockout mice under high-fat diet. Finally, we used C2BBe1, a subclone of Caco-2 cell, as a cell model to investigate the role of UFMylation in Coat Protein Complex II (COPII)-mediated lipid transport in enterocytes.

**Results:**

We serendipitously found that the combination of *Ufbp1* and *IRE1α* deficiencies led to dramatic accumulation of lipid droplets in the enterocytes, thereby impairing enterocyte function and causing significant growth retardation. Furthermore, we found that *Ufbp1* IEC-specific knockout mice were highly resistant to high-fat diet-induced hyperlipidemia. On the molecular level, we found that the components of the UFMylation pathway interacted with COPII complex and regulates the recruitment of COPII coat to ER-located lipoprotein.

**Conclusions:**

Our findings have established that the UFMylation pathway is a novel mediator of enterocyte lipid transport and a key partner of COPII-mediated trafficking.

## Highlights

• The UFMylation system is a novel regulator of intestinal lipid absorption and chylomicron secretion.
• Ufbp1 and IRE1α double deficiency causes severe accumulation of lipid droplets in enterocytes.
• Intestinal Ufbp1 deficiency protects mice from diet-induced hyperlipidemia.
• The UFMylation pathway interacts with COPII complex to facilitate its recruitment to ER exit site (ERES) of lipoproteins.

## 1 Introduction

Lipid absorption and transport in the small intestine is essential for nutrient uptake and systemic energy homeostasis [1]. Dysregulation of this process can lead to either hypolipidemia or hyperlipidemia and eventually cause metabolic diseases such as obesity and diabetes. Dietary lipids, primarily triglycerides (TGs), undergo enzymatic hydrolysis, and fatty acids (FAs) are absorbed by enterocytes via a combination of receptor-mediated active transport and passive diffuse mechanisms. After entering the enterocytes, FAs are re-esterified in the endoplasmic reticulum (ER), bound by apolipoproteins, and transported from the ER to Golgi apparatus, and finally form chylomicrons (CMs). Mature CMs are secreted via exocytosis and taken up by other tissues like liver, muscle and adipose tissues. The whole process of lipid metabolism in the enterocytes is tightly regulated, yet the precise underlying molecular mechanisms remain poorly understood [[2], [3], [4], [5], [6]].

One of the key events of lipid processing in the enterocytes is synthesis and transport of chylomicrons (CMs) [3,5,6]. CMs are complex particles containing multiple apolipoproteins, TGs, cholesterol and other lipid species [7]. CM assembly begins in the ER with the formation of pre-CM (pre-CM) complex in which TGs are loaded into apolipoprotein B48 (ApoB48)-containing lipoprotein particles with the help of microsomal triglyceride transfer protein (MTTP). Pre-CMs are subsequently transported from the ER via pre-CM transport vesicles (PCTV) and fuse with the Golgi apparatus in the coat protein complex II (COPII)-dependent manner. Within the Golgi apparatus, pre-CMs undergo lipidation and addition of ApoA1 protein to form mature CMs. Mature CMs are released from the Golgi apparatus, secreted into the lymphatic system and absorbed by other tissues and organs. Alternatively, TGs may also form cytoplasmic lipid droplets (LDs).

The Ufm1 (Ubiquitin fold modifier 1) conjugation system is a newly identified ubiquitin-like system that plays a critical role in ER homeostasis and protein quality control [8]. UFM1, the central modifier of the UFMylation pathway, is conjugated to target proteins through a series of biochemical reactions catalyzed by a set of Ufm1-specific enzymes, namely, Ufm1-activating E1 enzyme Uba5, Ufm1-conjugating E2 enzyme Ufc1, and Ufm1-specific E3 ligase(s) [9]. The only known Ufm1 E3 ligase consists of Ufl1 (Ufm1 ligase 1, also known as RCAD, NLBP, Maxer and KIAA0776) [[10], [11], [12], [13]] and its co-factor Ufbp1 (Ufl1 binding protein 1, also known as DDRGK1, Dashurin and C20orf116) [10,11,14]. Our previous studies have demonstrated that Ufl1 and Ufbp1 are essential for animal development and UFMylation of Ufm1 target proteins [[15], [16], [17]]. Recent studies indicate that UFMylation modulates lipid metabolism by maintaining ER stability and lipid synthesis [18]. In hepatocytes, Ufbp1 deficiency leads to lipid accumulation and increased susceptibility to high-fat diet (HFD)-induced liver injury [18] [19]. In intestinal epithelial cells, Ufbp1 deletion leads to the loss of Paneth and goblet cell function, exacerbating colitis susceptibility [17]. Yet, whether Ufbp1 is involved in lipid metabolism in the enterocytes remains unclear.

Here, we investigate the role of the UFMylation pathway and Ufbp1 protein in intestinal lipid metabolism. We found that UFMylation was critical for high-capacity lipid transport, and its deficiency led to accumulation of lipid droplets in the enterocytes and impaired secretion of lipoproteins into the plasma. Our work has demonstrated a novel function of UFMylation in lipid transport and may facilitate the development of new therapeutic strategy to combat metabolic diseases.

## 2 Materials and methods

### 2.1 Intestinal epithelial cell (IEC)-specific KO mice of *Ufbp1*, *PERK*, *IRE1α* and *DKO*

*Ufbp1*^f/f^ mice were generated in the Li lab and described previously [16]. *IRE1α*^f/f^ mice were obtained from Dr. Randal Kauffman's laboratory [20]. *PERK*^f/f^ mice (Jax # 023006) were purchased from Jackson Laboratories [21]. Villin-Cre transgenic mouse line was originally from Dr. Sylvie Robine's laboratory [22]. All mice were maintained on a C57BL/6J background. Genotyping was performed using PCR on tail DNA samples. The following PCR primers were utilized for the genotyping of the respective mouse strains: *Ufbp1*^f/f^ (5′- TAGTACTTGAAGTCTGGCTTGGTA-3′ and 5′-TAGTCAGGAACTGATGAGTGTCTC-3′), *IRE1α*^f/f^ (5′-CAGAGATGCTGAGTGAAGAC-3′ and 5′-ACAGTGGTTCCTGTGAAGGT-3′), *PERK*^f/f^ (5′-TTGCACTCTGGCTTTCACTC-3′ and 5′-AGGAGGAAGGTGGAATTTGG-3′), Villin-Cre (5′- GTG TGG GAC AGA GAA CAA ACC-3′ and 5′-ACA TCT TCA GGT TCT GCG GG-3′). Mice were housed under standard conditions with a 12-hour light/dark cycle and had access to food and water ad libitum. Diets were normal chow, containing 10% calories from fat or high-fat diets (HFD) containing 60% calories from fat (D12492, Research Diets, New Brunswick, NJ, USA). All experimental procedures were approved by the Institutional Animal Care and Use Committee (IACUC #2011-0314) at Augusta University and conducted in accordance with institutional guidelines. All mouse strains used in this study are listed in Key Resources (Table S8).

### 2.2 Chemical reagents, recombinant DNA and tissue culture cells

All chemicals were purchased from MiiliporeSigma (St. Louis, MO, USA), ThermoFisher Scientific (Waltham, MA, USA) and Cayman Chemical (Ann Arbor, MI, USA), and are listed in Key Resources (Table S5). HEK293T (ATCC # CRL-1573), C2BBe1 (ATCC # CRL-2102), U2OS (ATCC # HTB-96) and COS7 (ATCC # CRL-1651) cells were obtained from ATCC and cultured in DMEM supplemented with 10% fetal bovine serum (FBS), 1% penicillin-streptomycin and 2 mM l-glutamine at 37 °C in a humidified atmosphere with 5% CO2. human transferrin (0.01 mg/mL) was added in the complete medium for C2BBe1. Cells were passaged at 70–80% confluence using trypsin–EDTA and seeded at appropriate densities for subsequent experiments. For experimental treatments, cells were serum-starved for 12 h prior to the addition of specific reagents or drugs as indicated. All cell lines were regularly tested for mycoplasma contamination and authenticated using short tandem repeat (STR) profiling, and are listed in Key Resources (Table S7).

Molecular cloning was done using using E. Coli XL-1 blue cells (Table S4). All plasmids were prepared using E.Z.N.A.® Plasmid DNA Mini Kit I (Omega Bio-tek) (Table S6), and are listed in Key Resiurces (Table S9).

### 2.3 Preparation of lentiviruses expressing shRNAs and gRNAs

Lentiviral vectors encoding specific shRNAs and gRNAs were generated using the pLKO.1 (Addgene #10878) and lentiCRISPR V2 (Addgene #52961) vectors, respectively. Lentiviruses were produced in 293T packaging cells via polyethylenimine (PEI)-mediated transfection using three-plasmid system including psPAX2 packaging plasmid (Addgene #12260), pMD2.g envelop plasmid (Addgene #12259) and specific lentiviral plasmid, according to AU biosafety guideline (IBC #1004A). The sequences of shRNAs and gRNAs used in this study are listed below and in Table S2:

h Sec24D shRNA #1: CCCGTCTTTCAGAAGAAGGAA

h Sec24D shRNA #2: CGGATTCACAATCTTGGCTTA

hUfbp1 gRNA: GTAGCGGCGGCTCTGCTAGT

hIRE1α gRNA: CTTGTTGTTTGTGTCAACGC

hUfm1 gRNA #1: TCACGCTGACGTCGGACCCA

hUfm1 gRNA #2: CTTTAAGATCACGCTGACGT

hUba5 gRNA #1: TCCCGAGGAGCGGCGACGGA

hUba5 gRNA #1: GCTGGAGCGGGAACTTGCCC.

### 2.4 Generation of shRNA and gRNA-mediated knockdown cell lines

For shRNA and CRISPR/Cas9-mediated gene knockdown, C2BBe1 or COS7 cells were infected with lentiviruses expressing either control or gene-specific shRNA/gRNA for 48 h and then selected with puromycin (1.5 μg/ml) for another 48 h. Knockdown efficiency was evaluated by immunoblotting.

### 2.5 ApoB48-GFP secretion assay in COS7 cells

ApoB48-GFP trafficking assay was performed according to Walsh et al. [23] with a modification. Human MTP-Flag fusion gene was amplified from hMTP-Flag plasmid (Addgene #138335) [24] using primers: 5′-AGGATCCATGATTCTTCTTGCTGTGCTT-3′ and 5′-AGCGGCCGCTCACTTGTCGTCATCGTCCTT-3′, then subcloned into lentiviral vector pCDH-puro plasmid. COS7 cells were infected with lentivirus expressing hMTP-Flag to establish a cell line stably expressing hMTP-Flag protein. These cells were then infected with lentiviruses expressing *Ufbp1* and *IRE1α* gRNAs to establish stable knockdown lines. Subsequently, cells were transfected with ApoB48-GFP expression plasmid (Addgene #138334) [23]. At 24 h post-transfection, cells were trypsinized and replated. After 24-hour incubation, cells were treated with oleic acid (OA) in DMEM without FBS. After overnight incubation, both culture media and cell lysates were harvested and subjected to immunoblotting and chemiluminescence detection. Digital images were acquired by ChemiDoc MP imaging system (Bio-Rad, Hercules, California, USA), and band intensity was quantitated with ImageLab software (Bio-Rad).

### 2.6 Immunoprecipitation

Immunoprecipitation assays were performed in HEK293T cells. Briefly, HEK293T cells were cultured in DMEM supplemented with 10% FBS and transfected with the indicated expression plasmid using polyethyleneimine (PEI) according to the manufacturer's instruction. 24 h after transfection, cells were washed twice with ice-cold PBS and lysed on ice for 30 min in IP lysis buffer (50 mM Tris–HCl, pH 7.4, 150 mM NaCl, 1 mM EDTA, 1% Nonidet P-40) supplemented with protease inhibitor cocktail. Cell lysates were clarified by centrifugation at 12,000×*g* for 10 min at 4 °C, and the supernatants were incubated with anti-Flag M2 beads at 4 °C overnight with gentle rotation. The beads were washed four times with lysis buffer and bound proteins were eluted using 0.1M glycine (pH 2.5). Eluates were immediately neutralized with 1 M Tris–HCl (pH 8.0), followed by boiling in SDS sample buffer.

### 2.7 Immunoblotting and analysis

Mice were euthanized after the indicated treatment, and the small intestine were removed and rinsed twice with ice-cold PBS to remove luminal contents. The tissues were then cut into small pieces and incubated in ice-cold EDTA (0.5 mM) PBS buffer on a shaker for 30 min. The resulting villus fraction was collected by centrifugation (1000 rpm, 5 min), and the pellet was resuspended in RIPA buffer (50 mM Tris–HCl, pH 7.4, 150 mM NaCl, 1% Triton X-100, 1% sodium deoxycholate, and 0.1% SDS) plus protease inhibitor cocktail (Roche, Cat#4693116001) for protein extraction. Plasma samples were collected by centrifugation of whole blood at 3000 rpm, 5 min and subsequently subjected to protein extraction for immunoblotting analysis. Protein concentrations were determined using the BCA assay (ThermoFisher, Cat#23232). Equal amounts of protein were separated by SDS-PAGE and transferred to PVDF membranes. Membranes were blocked with 5% non-fat dry milk in TBST for 1 h at room temperature, followed by overnight incubation at 4 °C with primary antibodies diluted in 1% BSA in TBST (20 mM Tris, pH 7.4, 150 mM NaCl and 0.1% Tween-20). After washing, membranes were incubated with HRP-conjugated secondary antibodies for 1 h at room temperature. Protein bands were visualized using an enhanced chemiluminescence (ECL) detection system (Clarity Western ECL Substrate, Bio-Rad) and acquired with a Bio-Rad ChemiDoc MP Imaging System. Densitometric analysis was performed using ImageLab software (Bio-Rad) and ImageJ softwares. All antibodies and reagents used are listed in Key Resources (Table S3).

### 2.8 Immunofluorescence

Paraffin-embedded tissue sections were washed with xylene to remove the wax and then rehydrated through a series of graded alcohol solutions and water. Antigen retrieval was achieved by incubation in citrate-based Antigen Unmasking solution (H-3300, Vector Laboratoires, Inc., Burlingame, California, USA) at 95 °C for 20 min. Sections were then incubated with indicated primary antibodies overnight at 4 °C, followed by specific fluorophore-conjugated secondary antibodies. Epifluorescence images were acquired by Zeiss Observer D1 with AxioVision 4.8 software (Carl Zeiss Microscopy GmbH, Jena, Germany).

For confocal imaging, C2BBe1 cells cultured at the top of glass coverslips were fixed in 4% paraformaldehyde and permeabilized with 0.1% Triton X-100. Primary antibodies were applied overnight at 4 °C, followed by washing and incubation with fluorophore-conjugated secondary antibodies for 1 h at room temperature. Nuclei were counterstained with DAPI. Confocal images were acquired by Leica Stellaris 5 confocal microscope with 63x lens and analyzed with Leica LAX S software (Leica Microsystems, Inc., Bannockburn, IL, USA). Confocal images were analyzed using ImageJ, and the Pearson's correlation coefficient was calculated with JACoP (Just Another Colocalization Plugin).

### 2.9 Transmission electron microscopy

Transmission Electron Microscopy (TEM) was performed by the EM Core at Augusta University using established procedures. Tissues were fixed in 2.5% glutaraldehyde in 0.1 M cacodylate buffer (pH 7.4) overnight at 4 °C. After washing in cacodylate buffer, samples were post-fixed in 1% osmium tetroxide for 1 h at room temperature, dehydrated through a graded ethanol series, and embedded in Epon resin. Ultrathin sections (70 nm) were cut using an ultramicrotome (Leica UC7) and collected on copper grids. Sections were stained with 2% uranyl acetate for 15 min and lead citrate for 10 min. Images were acquired using a transmission electron microscope (JEOL JEM-1400) at an accelerating voltage of 80 kV. Digital images were captured with a Gatan CCD camera. All reagents and protocols are detailed in the Key Resources.

### 2.10 H&E and PAS/Alcian Blue staining

Hematoxylin and Eosin (H&E), as well as PAS/Alcian blue staining, were conducted by the Histology Core at Augusta University following their standard protocols. Paraffin-embedded tissue sections (5 μm) were deparaffinized in xylene and rehydrated through a graded ethanol series. For H&E staining, sections were stained with hematoxylin, differentiated in 1% acid alcohol, blued in 0.2% ammonia water, counterstained with eosin, dehydrated, cleared in xylene, and mounted with synthetic resin. For PAS/Alcian Blue staining, sections were oxidized in 0.5% periodic acid, treated with Schiff's reagent, washed, counterstained with hematoxylin, blued, dehydrated, cleared in xylene, and mounted with synthetic resin. Images for both staining methods were captured using a light microscope (Zeiss Axio Observer). All reagents and protocols are listed in the Key Resources.

### 2.11 Oli red O staining and measurement

Frozen tissue sections (10 μm) were fixed in 4% paraformaldehyde at 4 °C for 24 h. The samples were subsequently cryoprotected in 30% sucrose for 12 h and embedded in Tissue-Tek O.C.T. Compound (Sakura Finetek, Torrance, CA, USA). Serial sections (5 μM) were stained with freshly prepared Oil Red O solution for 15 min at room temperature, rinsed in 60% isopropanol, and counterstained with hematoxylin. Sections and coverslips were mounted with aqueous mounting medium. Images were acquired with Keyence BZ-X700 fluorescent microscope with its corresponding software (Keyence America, Itasca, IL, USA).

For C2BBe1 cells, control and knockdown cells were plated on 12-well plates and cultured for 36 h, followed by overnight treatment with 0.5 mM OA or BSA. Cells were fixed in 4% paraformaldehyde for 15 min at room temperature, washed and subsequently stained with freshly prepared Oil Red O solution for 15 min at room temperature and rinsed in 60% isopropanol. Images were acquired with Keyence BZ-X700 fluorescent microscope with its corresponding software (Keyence America, Itasca, IL, USA). To quantify Oil Red O content, stained lipids were extracted with dimethyl sulfoxide (DMSO). After gentle shaking at room temperature for 5 min, absorbance at 510 nm was measured at 510 nm using Nanodrop 2000 (ThermoFisher Scientific). Cells were further lysed to extract total protein, and protein concentrations were determined by BCA assay (ThermoFisher Scientific, Cat#23232) (Table S6).

### 2.12 Glucose tolerance and insulin tolerance tests

For oral glucose tolerance test (OGTT), 25-week-old mice were fasted overnight and administered glucose by oral gavage at a dose of 6 g/kg body weight. Blood glucose levels were measured at the indicated time points using a glucometer (IMDK Blood Glucose Monitor Kit). For insulin tolerance test (ITT), 26-week-old mice were fasted for 6 h and received an intraperitoneally injection of insulin (1.5 U/kg body weight). Blood samples were collected from the tail vein at specified intervals and glucose were determined using the glucometer (Metene) (Table S6).

### 2.13 Measurement of lipid content

Commercially available assay kits were used to measure plasma triglyceride (NBP3-24540, NOVUS Biologicals, Centennial, CO, USA) and total cholesterol (NBP3-25838, NOVUS Biologicals) according to the manufacturer's instructions. For hepatic TG and TC quantification, liver tissues were homogenized in methanol at a ratio of 9:1 (methanol volume: tissue weight). The homogenates were then centrifuged at 10,000 × *g* for 10 min at 4 °C. The resulting supernatants were collected and kept on ice until analysis. Assays kits used in this study are listed in Key Resources (Table S6).

### 2.14 Quantitative real-time PCR

Total RNA was isolated with the EZNA HP Total RNA Isolation kit (Omega BIO-TEK, Norcross, GA) (Table S6), and then reversely transcribed with the High-Capacity cDNA Reverse Transcription kit according to the manufacturer's instruction (ThermoFisher Scientific) (Table S6). Quantitative RT-PCR was performed using HotStart 2x Green qPCR Master MIx (APExBIO, Boston, MA, USA) (Table S6) with 40 cycles of 95 °C for 15 s and 60 °C for 1 min on StepOnePlus Real-Time PCR System (ThermoFisher Scientific). The results were analyzed by StepOne Software (Version 2.1, Life Technologies). The relative expression of each transcript was normalized to murine GAPDH by using the 2ˆ (-delta delta Ct) method. The primers used in this study were described in Key Resources (Table S1)

### 2.15 RNA sequencing (RNA-seq) and bioinformatics analysis

Total RNA was isolated with the EZNA HP Total RNA Isolation kit (Omega BIO-TEK, Norcross, GA). RNA integrity was assessed using an Agilent 2100 Bioanalyzer, and samples with an RNA integrity number (RIN) ≥ 7.0 were used for library preparation. RNA sequencing and bioinformatic analysis was provided by Novogene (Sacramento, CA, USA). Raw data were obtained by Illumina platform. After data filtering, the genes were mapped to the genome with STAR software, and quantification was determined by HTSeq software. DESeq2 was used for differential analysis, and ClusterProfiler was used for enrichment analysis (GO, KEGG and Reactome). Visualization of DEGs was conducted via heatmaps (pheatmap), volcano plots (ggplot2), and principal component analysis (PCA) to assess sample clustering and biological variation.

### 2.16 Statistical analysis

Statistical analyses were performed using GraphPad Prism 10 and IBM SPSS V22 softwares. Data are presented as mean ± SEM. Two-group comparisons were conducted using an independent Samples *t*-test. For multiple group comparisons, one-way ANOVA followed by Tukey's post hoc test was used. A *p*-value of less than 0.05 was considered statistically significant. Detailed statistical methods and n values are provided in the figure legends. All softwares used in this study are listed in Key resources (Table S10)

## 3 Results

### 3.1 *Ufbp1* and *IRE1α* double knockout impaired postnatal growth

As previously reported, intestinal epithelial cell (IEC)-specific *Ufbp1* knockout (*Ufbp1*^Δ/ΔIEC^) mice exhibited loss of Paneth and goblet cells in the intestine, and *Ufbp1* deficiency led to activation of Unfolded Protein Response (UPR) and up-regulation of UPR-related genes [17]. To investigate the underlying mechanism, we attempted to determine if over-activation of PERK and IRE1α, two branches of UPR, causes the loss of secretory cells in *Ufbp1*^Δ/ΔIEC^ intestine. First, we created IEC-specific knockout of *PERK* (*PERK*^Δ/ΔIEC^) and double-knockout (DKO) mice of *Ufbp1* and *PERK* (*Ufbp1*^Δ/ΔIEC^;*PERK*^Δ/ΔIEC^) and compared them to wild-type and *Ufbp1*^Δ/ΔIEC^ mice (Supplemental Fig. S1A). Knockout of *PERK* alone did not affect the intestinal epithelium (Supplemental Figs. S1B and 1C). Additionally, *PERK* knockout failed to rescue the loss of Paneth and goblet cells caused by *Ufbp1* deficiency (Supplemental Fig. S1D and S1E, compare *Ufbp1*^Δ/ΔIEC^ and *Ufbp1*^Δ/ΔIEC^;*PERK*^Δ/ΔIEC^ mice), suggesting that PERK activation does not contribute to the loss of *Ufbp1* deficient exocrine cells.

**Figure 1:**
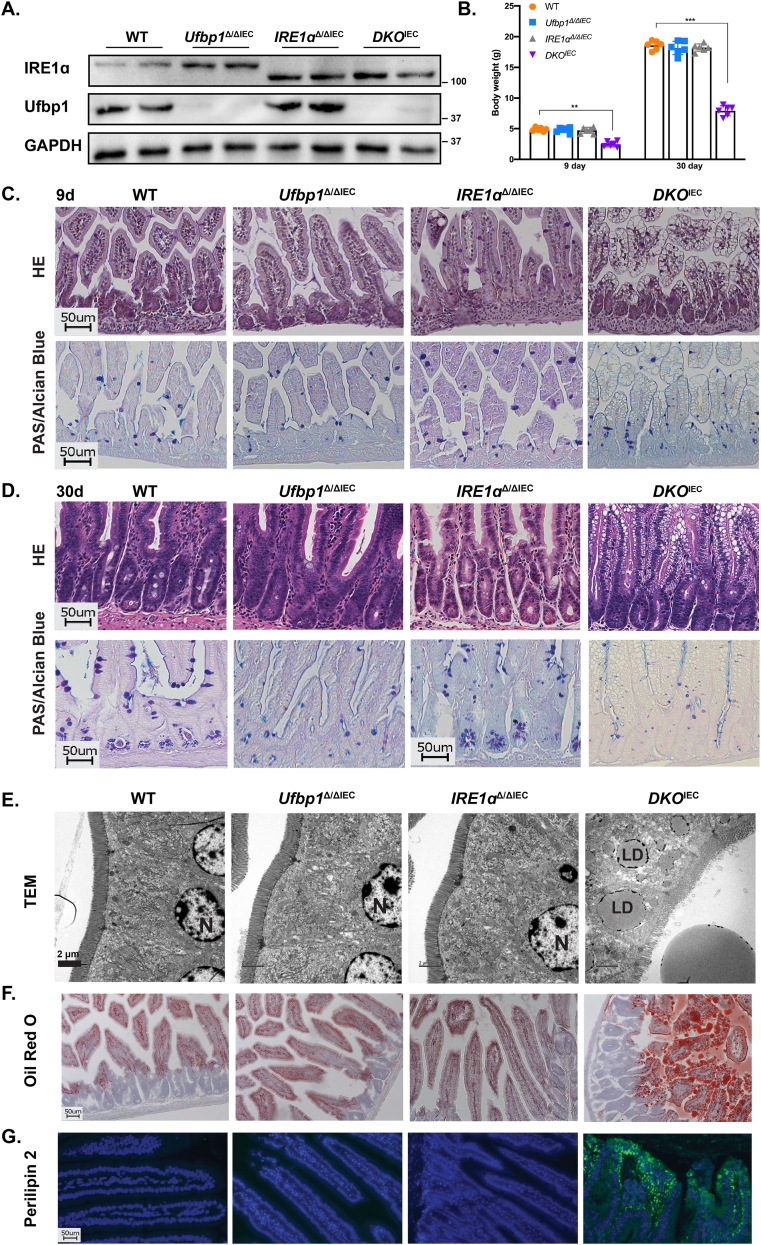
**Growth retardation of *Ufbp1*^Δ/ΔIEC^;*IRE1α*^Δ/ΔIEC^ DKO mice.** (A) Protein levels of Ufbp1 and IRE1α in intestine tissues. Western blot analysis of intestinal tissue lysates from WT, *Ufbp1*^Δ/ΔIEC^, *IRE1α*^Δ/ΔIEC^, and *DKO*^IEC^ mice. Blots shown are representative of two biological replicates. Cre-mediated deletion of floxed exon 16 and 17 in murine *IRE1α* gene resulted in a smaller IRE1α protein with a truncated endonuclease domain and inactive enzyme. (B) Body weight of WT, *Ufbp1*^Δ/ΔIEC^, *IRE1α*^Δ/ΔIEC^, and *DKO*^IEC^ mice at post-natal 9-day and 30-day. Data are represented as mean ± SEM (n = 6 mice per group). Statistical significance was determined using one-way ANOVA with Tukey's post hoc test. ∗∗*P* < 0.01, ∗∗∗*P* < 0.001. (C and D) Representative H&E and PAS/Alcian Blue staining of proximal small intestines from 9-day and 30-day WT, *Ufbp1*^Δ/ΔIEC^, *IRE1α*^Δ/ΔIEC^, and *DKO*^IEC^ mice. Scale bar, 50 μm. (E) Representative TEM images of the small intestines of WT, *Ufbp1*^Δ/ΔIEC^, *IRE1α*^Δ/ΔIEC^ and *DKO*^IEC^ mice. Lipid droplets were markers as “LD”, and nucleus was marked as “N”. (F) Oil-Red-O staining of the small intestines of WT, *Ufbp1*^Δ/ΔIEC^, *IRE1α*^Δ/ΔIEC^ and *DKO*^IEC^ mice. (G) Perilipin 2 immunostaining of the small intestines of WT, *Ufbp1*^Δ/ΔIEC^, *IRE1α*^Δ/ΔIEC^ and *DKO*^IEC^ mice.

Next, we generated *Ufbp1* and *IRE1α* DKO mice (*Ufbp1*^Δ/ΔIEC^;*IRE1α*^Δ/ΔIEC^, also referred as to *DKO*^IEC^) to investigate the role of IRE1α in the loss of *Ufbp1* deficient exocrine cells (Supplemental Fig. S2A). Cre-mediated deletion of floxed exon 16 and 17 in murine *IRE1α* gene results in a smaller IRE1α protein with a truncated endonuclease domain, thereby inactivating IRE1a signaling [20]. Tissue-specific deletion of *Ufbp1* and *IRE1α* was confirmed by genotyping and Western blot analysis (Figure 1A and Supplemental Fig. S2B). Both *Ufbp1*^Δ/ΔIEC^ and *IRE1α*^Δ/ΔIEC^ mice were born at Mendelian ratios and showed no apparent defects at weaning and postnatal development. In contrast, *Ufbp1*^Δ/ΔIEC^;*IRE1α*^Δ/ΔIEC^ DKO mice exhibited postnatal growth retardation, body weight reduction, and frequent mortality after weaning (Figure 1B and Supplemental Fig. S2C). The body weight of *DKO*^IEC^ mice was significantly lower than that of wild-type (WT) and single knockout mice at both postnatal day 9 and day 30 (Fig.1B). To further characterize the intestinal architecture across different mouse models, we performed hematoxylin & eosin (H&E) and Periodic Acid-Schiff (PAS)/Alcian blue staining of small intestinal tissues harvested on postnatal day 9 (P9) and day 30 (P30). Compared to WT intestine, *IRE1α*^Δ/ΔIEC^ intestine exhibited no gross structural abnormality (Figure 1C,D). As previously shown, there was a significant reduction of Paneth and goblet cells in *Ufbp1*^Δ/ΔIEC^ intestine (P30), and this phenotype was not reversed by *IRE1α* knockout (compare *Ufbp1*^Δ/ΔIEC^ mice and *Ufbp1*^Δ/ΔIEC^;*IRE1α*^Δ/ΔIEC^ DKO mice) (Supplemental Figs. S2D and 2E). This result suggests that IRE1α activation is not responsible for *Ufbp1* deficiency-induced loss of exocrine cells. Unexpectedly, the enterocytes in *Ufbp1*^Δ/ΔIEC^;*IRE1α*^Δ/ΔIEC^ DKO intestine contained many vacuole-like structures (Fig.1C and 1D, *DKO*^IEC^ panels), indicating that knockout of both *Ufbp1* and *IRE1α* may impair the development and/or function of the enterocytes, thereby resulting in malnutrition and growth retardation of *Ufbp1*^Δ/ΔIEC^;*IRE1α*^Δ/ΔIEC^ DKO mice.

### 3.2 DKO of *Ufbp1* and *IRE1α* led to massive accumulation of lipid droplets in the enterocytes

The abnormal enterocytes in *DKO*^IEC^ intestine prompted us to further determine the nature of the vacuole-like structures. Ultrastructural analysis using transmission electron microscopy (TEM) showed that, compared to the enterocytes of WT and single knockout mice, the enterocytes of *DKO*^IEC^ intestine contained extensive intracellular structures that appeared to be lipid droplets (LDs) (Fig.1E). To confirm the accumulation of LDs in *DKO*^IEC^ enterocytes, we performed Oil Red O staining that specifically detects neutral lipid deposits. As shown in Figure 1F, there were no significant differences between *Ufbp1*^Δ/ΔIEC^, *IRE1α*^Δ/ΔIEC^ mice and WT controls. In contrast, *DKO*^IEC^ mice exhibited markedly enlarged lipid droplets and severe lipid accumulation (Fig.1F). Comparative analysis of H&E and Oil Red O staining in WT and *DKO*^IEC^ mice revealed that the vacuolar structures observed in H&E staining corresponded to LDs, confirming that the vacuolization in *DKO*^IEC^ enterocytes was indeed a result of LD deposition in the intestinal epithelium (Supplemental Fig. S2F). Consistently, immunostaining of LD-associated protein Perilipin 2 further confirmed the pronounced LD accumulation in *DKO*^IEC^ enterocytes (Fig.1G). In addition to lipid accumulation, *DKO*^IEC^ enterocytes exhibited disorganized and shortened microvilli (Supplemental Figs. S2G and 2H), which may lead to impaired enterocyte function and malnutrition of postnatal pups. Together, these results strongly suggest the involvement of Ufbp1 in lipid metabolism in the enterocytes.

### 3.3 *Ufbp1* and *IRE1α* knockout affected the genes related to lipid metabolism

To gain mechanistic insight into the molecular alterations in the small intestine, we performed RNA-seq analysis on different mouse models. Correlation analysis and principal component analysis (PCA) revealed distinct differences among WT, *Ufbp1*^Δ/ΔIEC^, *IRE1α*^Δ/ΔIEC^ and *DKO*^IEC^ mice (Supplemental Fig. S3A and S3B). Clustering and Venn analyses were performed to depict the expression patterns of differentially expressed genes (DEGs) across the four groups of mice (Supplemental Fig. S3C and S3D). Additionally, volcano plots provided the numbers of upregulated and downregulated genes between groups (Supplemental Fig. S3E). In the *Ufbp1*^Δ/ΔIEC^ vs WT group, DEGs included 2688 upregulated and 2564 downregulated genes (Supplemental Fig. S3E, 2-fold change with p_adj_ < 0.05). In the *DKO*^IEC^ vs WT group, DEGs includes 1824 upregulates and 1745 downregulated genes (Supplemental Fig. S3E). In contrast, *IRE1α* knockout exerted a modest effect on gene expression (Supplemental Fig. S3E).

**Figure 2:**
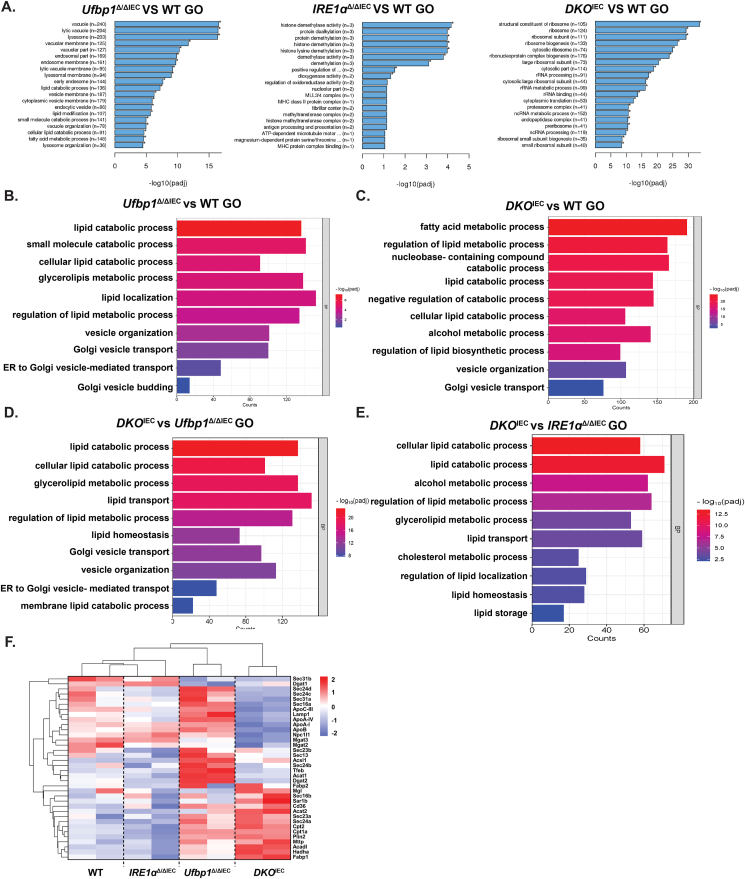
***Ufbp1* and *IRE1α* knockout affected expression of the genes related to lipid metabolism.** (A) GO enrichment analysis of DEGs in the small intestines of *Ufbp1*^Δ/ΔIEC^, *IRE1α*^Δ/ΔIEC^, and *DKO*^IEC^ vs WT. Bar plots show the top enriched biological process (BP) terms in each group. (B and C) GO (BP) enrichment analysis of lipid metabolism-related DEGs among WT, *Ufbp1*^Δ/ΔIEC^, and *DKO*^IEC^ intestines. (D and E) GO (BP) enrichment analysis of lipid metabolism-related DEGs of *DKO*^IEC^ vs single KO mice. (F) Heatmap of lipid metabolism-related DEGs among the four animal groups. Hierarchical clustering revealed distinct transcriptional signatures, with upregulated (red) and downregulated (blue) genes clearly separated among animal groups. Key genes involved in lipid transport, lipoprotein assembly, and ER-associated lipid metabolism exhibited significant expression changes. Z-score normalization was applied to gene expression values, and clustering was performed using Euclidean distance.

Gene Ontology (GO) enrichment analyses of DEGs revealed significant changes in protein biogenesis and lipid metabolism-related pathways. In the *Ufbp1*^Δ/ΔIEC^ vs WT group, GO terms were associated with vacuole, lysosome, and lipid metabolism were significantly enriched (Figure 2A). In comparison, ribosomal biogenesis was predominantly enriched in the *DKO*^IEC^ vs WT groups (Fig.2A). To further examine lipid metabolism-related changes, we conducted a focused GO enrichment analysis restricted to lipid-associated terms. The results revealed that both *Ufbp1*^Δ/ΔIEC^ and *DKO*^IEC^ mice displayed enrichment in biological processes related to lipid metabolism compared with WT (Figure 2B,C). Importantly, *DKO*^IEC^ mice showed distinct alterations in lipid catabolic process and lipid transport-related functions compared with *Ufbp1*^Δ/ΔIEC^ and *IRE1α*^Δ/ΔIEC^ mice (Figure 2D,E). Specifically, the genes involved in lipid trafficking, including ApoA, B, C genes and Sec24 genes, were upregulated in *Ufbp1*^Δ/ΔIEC^ mice, and their up-regulation was abolished in *DKO*^IEC^ mice (Figure 2F and Supplemental Fig. S4A). These results indicate that LD accumulation in *DKO*^IEC^ enterocytes may be caused by impaired lipid transport.

### 3.4 *Ufbp1* and *IRE1α* deficiency promoted oleic acid (OA)-induced lipid accumulation in C2BBe1 cells

**Figure 3:**
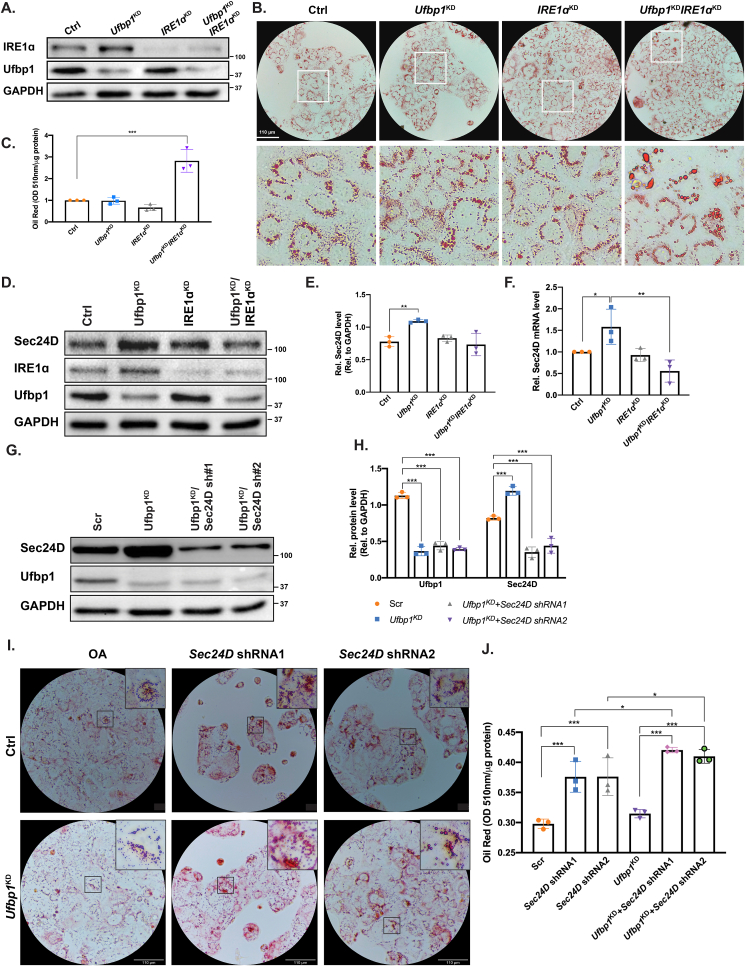
***Ufbp1* and *IRE1α* deficiency caused oleic acid (OA)-induced lipid accumulation in C2BBe1 cells.** (A) Representative Western blot images showing protein levels of Ufbp1 and IRE1α in vector control, *Ufbp1*^KD^, *IRE1α*^KD^, and *Ufbp1*^KD^*IRE1α*^KD^ C2BBe1 cells. C2BBe1 cells were infected with lentiviral vectors expression Ufbp1 and IRE1α gRNAs for 48 h, followed by puromycin (1.5 μg/ml) selection for another 48 h. Cells were further cultured for 24–48 h and collected for immunoblotting analysis. (B) Representative images of Oil Red O staining in control, *Ufbp1*^KD^, *IRE1α*^KD^, and *Ufbp1*^KD^*IRE1α*^KD^ C2BBe1 cells after 0.5 mM OA treatment for 8 h. Lipid droplets are stained as red. Scale bar, 110 μm. (C) Quantification of Oil Red O intensity. Dimethyl sulfoxide (DMSO) was added to each sample. After shaking at room temperature for 5 min, the density of samples was measured at 510 nm on Nanodrop 2000. Data represent mean ± SEM (n = 3), analyzed by one-way ANOVA with Tukey's post hoc test. ∗∗∗*P* < 0.001. (D) Representative Western blot images showing protein levels of Ufbp1, IRE1α and Sec24D in control, *Ufbp1*^KD^, *IRE1α*^KD^, and *Ufbp1*^KD^*IRE1α*^KD^ C2BBe1 cells. (E) Quantitation of Sec24D protein level from three independent experiments. GAPDH served as a loading control. Data are presented as mean ± SEM (n = 3). Statistical significance was determined using one-way ANOVA with Tukey's post hoc test. ∗*P* < 0.05; ∗∗*P* < 0.01. (F) Quantification of *Sec24D* mRNA level normalized to GAPDH. Data are presented as mean ± SEM (n = 3). Statistical significance was determined using one-way ANOVA with Tukey's post hoc test. ∗*P* < 0.05; ∗∗*P* < 0.01. (G) Representative Western blot images showing protein levels of Ufbp1 and Sec24D in control, *Ufbp1*^KD^, *Ufbp1*^KD^ combined with Sec24D shRNAs C2BBe1 cells. (H) Quantitation of Ufbp1 and Sec24D protein levels from three independent experiments. GAPDH served as a loading control. Data are presented as mean ± SEM (n = 3). Statistical significance was determined using one-way ANOVA with Tukey's post hoc test. ∗∗∗*P* < 0.001. (I) Oil Red O staining of control, *Ufbp1*^KD^, *Ufbp1*^KD^ combined with Sec24D shRNAs, C2BBe1 cells after 8-hour treatment of 0.5 mM OA. Lipid droplets are stained red. Scale bar, 110 μm. (J) Quantification of Oil Red O intensity. Data represent mean ± SEM (n = 3) from three independent experiments and analyzed by one-way ANOVA with Tukey's post hoc test. ∗*P* < 0.05; ∗∗∗*P* < 0.001.

To further validate our findings at the cellular level, we took advantage of C2BBe1, a subclone of colorectal cancer cell line Caco-2 that has been widely used as an enterocyte model to study absorption and barrier function of intestinal epithelium [25]. We generated *Ufbp1*, *IRE1α*, and double knockdown (KD) cell lines (Figure 3A). Notably, upon 0.5 mM OA treatment, double KD cells displayed pronounced lipid accumulation with enlarged lipid droplets, while single KD of either *Ufbp1* or *IRE1α* had no significant effect on lipid accumulation (Figure 3B, C and Supplemental Fig. S5). This result recapitulated the phenotype of *DKO*^IEC^ enterocytes and further confirmed the role of Ufbp1 and IRE1α in lipid metabolism in enterocytes.

### 3.5 *Sec24D* knockdown led to lipid accumulation in C2BBe1 cells

ER-to-Golgi transport of pre-CMs is dependent on COPII complex [5] [6] [26] [27]. Our bulk RNA-seq data showed that expression of multiple COPII genes, including *Sec13*, *Sec23A* and *B*, *Sec24A*, *C* and *D*, and *Sec31B*, were altered in *Ufbp1*^Δ/ΔIEC^ and *DKO*^IEC^ intestines (Figure 2F and Supplemental Fig. S4A). Among them, *Sec24D* level was significantly elevated in *Ufbp1*^Δ/ΔIEC^, and the elevation was blocked by *IRE1α* knockout (Supplemental Fig. S4A, panel Sec24D). We preformed quantitative RT-PCR and immunoblotting analyses to validate our RNA-seq data. In agreement with our previous study, *Ufbp1* knockout up-regulated *IRE1α* and *Sec24D* expression (Supplemental Figs. S4B and 4C). Interestingly, the increase of *Sec24D* expression induced by *Ufbp1* knockout was inhibited by *IRE1α* deletion (Supplemental Figs. S4B and 4C). This result was consistent with the previous study showing that *Sec24D* expression is regulated by IRE1α/Xbp-1 signaling [28]. We also examined the protein and mRNA expression levels of Sec24D in C2BBe1 cells. Consistent with *in vivo* findings, *Ufbp1* knockdown caused increased expression of Sec24D, and the increase was blocked by *IRE1α* knockdown (Figure 3D,E and 3F). To investigate the role of Sec24D in lipid metabolism, we examined lipid accumulation in *Sec24D* knockdown cells. Oil Red O staining revealed that *Sec24D* knockdown resulted in enlarged lipid droplets and increased lipid accumulation compared to control cells (Figure 3G–J). We further tested the effect of dual knockdown of *Ufbp1* and *Sec24D*. Interestingly, Oil Red O staining showed that *Ufbp1* knockdown further exacerbated lipid droplet enlargement and accumulation in *Sec24D* knockdown cells (Figure 3G–J). This result suggests that Ufbp1 may modulate COPII-dependent lipid transport in enterocytes.

### 3.6 Deficiency of *Ufbp1* and *IRE1α* led to impaired apolipoprotein secretion

**Figure 4:**
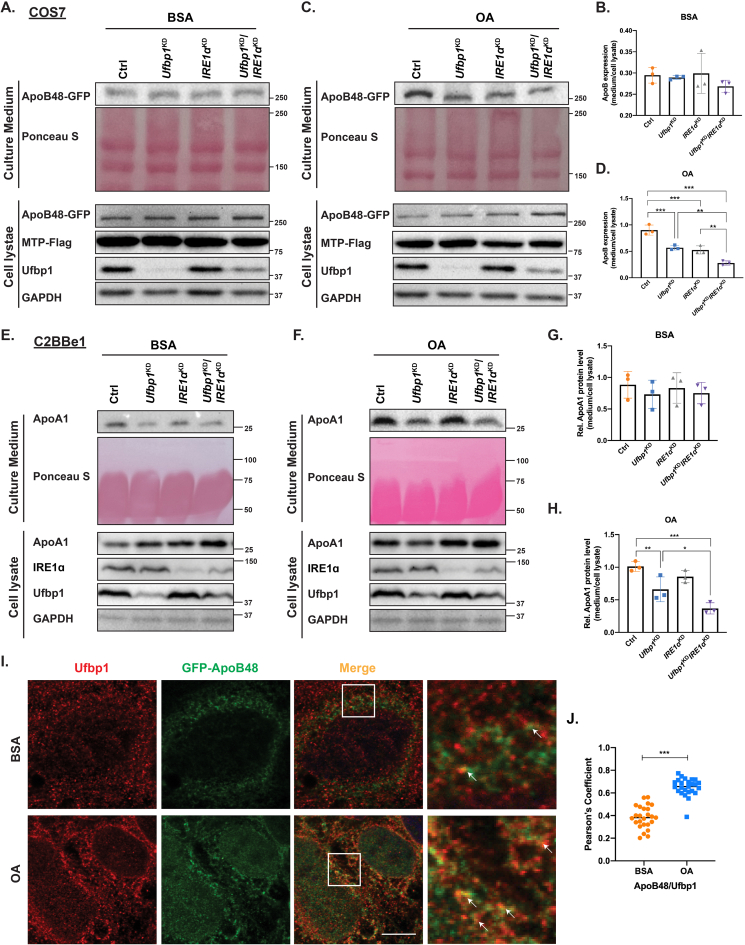
**Deficiency of Ufbp1 and IRE1α led to impaired apolipoprotein trafficking** (A) ApoB48-GFP level in culture media and cell lysates in BSA (control)-treated COS7 cells with stable expression of MTP-Flag protein and knockdown of Ufbp1 and IRE1α proteins. ApoB48-GFP plasmid were transiently transfected into COS7 cells. After 36h, cells were incubated with either BSA- or OA (complexed with BSA)-containing media for 24 h. Both culture medium and cell lysate were subjected to immunoblotting. (B) Quantitation of the relative level of ApoB48-GFP in the medium vs cell from three independent experiments of BSA-treated cells. Ponceau S and GAPDH served as loading controls for culture medium and cell lysate, respectively. Data are presented as mean ± SEM (n = 3). Statistical significance was determined using one-way ANOVA with Tukey's post hoc test. ∗∗*P* < 0.01, ∗∗∗*P* < 0.001. (C) ApoB48-GFP level in culture media and cell lysates in OA-treated COS7 cells. (D) Quantitation of the relative levels of ApoB48-GFP in the medium and cell from three independent experiments of OA-treated cells. Data are presented as mean ± SEM (n = 3). Statistical significance was determined using one-way ANOVA with Tukey's post hoc test. ∗∗*P* < 0.01, ∗∗∗*P* < 0.001. (E) Secretion of endogenous ApoA1 in BSA-treated C2BBe1 cells. Control, *Ufbp1*^KD^, *IRE1α*^KD^, and *Ufbp1*^KD^*IRE1α*^KD^ C2Bbe1 cells were plated overnight, cells were incubated with BSA (control) or 0.5 mM OA complexed with BSA for 24 h. Media and cells were collected and subjected to immunoblotting analysis. (F) Secretion of endogenous ApoA1 in OA-treated C2BBe1 cells. (G) Quantitation of the relative level of ApoA1 in the medium vs cell from three independent experiments of BSA-treated cells. (H) Quantitation of the relative level of ApoA1 in the medium vs cell from three independent experiments of OA-treated cells. ApoA1 levels measured in the cell lysate and media. Ponceau S and GAPDH served as loading controls. Data are presented as mean ± SEM (n = 3). Statistical significance was determined using one-way ANOVA with Tukey's post hoc test. ∗*P* < 0.05, ∗∗*P* < 0.01, ∗∗∗*P* < 0.001. (I) Co-localization of ApoB48-GFP and endogenous Ufbp1 in BSA- or OA-treated C2BBe1 cells. C2BBe1 cells were plated on glass coverslips overnight and then transiently transfected with ApoB48-GFP plasmid. After 24-hour incubation, cells were incubated with BSA or OA-containing medium for 8 h. Cells were then fixed and subjected to Ufbp1 immunostaining. (J) Pearson's coefficients of ApoB48-GFP and Ufbp1 co-localization in BSA- and OA-treated cells. Data were analyzed with Image J software and presented as mean ± SEM (n = 25 cells). Statistical significance was determined using Independent-Samples *t* test. ∗∗∗*P* < 0.001.

To further investigate the role of Ufbp1 and IRE1α in lipid trafficking, we took advantage of established COS-7 cell model in which ApoB48-GFP secretion is responsive to lipid upload [23]. ApoB48 is one of key lipoproteins that are critical for chylomicron formation and transportation in the enterocytes. When Apo48-GFP was transiently expressed in COS-7 cells stably expressing lipid transfer protein MTP-Flag, there was a basal level of secretion of ApoB48-GFP under normal culture medium. When OA was added into the medium, more ApoB48-GFP was found in the medium (Supplemental Fig. S6A), and this increase was inhibited by brefeldin A (BFA), an inhibitor of ER to Golgi apparatus trafficking (Supplemental Fig. S6B). We next examined ApoB48-GFP secretion in *Ufbp1* and *IRE1α* knockdown cells. Knockdown of either *Ufbp1* or *IRE1α* had minimal effect on basal level of extracellular ApoB48-GFP in the control medium (BSA) (Figure 4A,B). Interestingly, a significant decrease in extracellular ApoB48 levels and concomitant accumulation of intracellular ApoB48 was observed in all three knockdown cells when cells were treated with OA (Figure 4C,D). We also examined OA-induced secretion of endogenous apolipoproteins in C2BBe1 cells. While knockdown of *Ufbp1* or *IRE1α* or both did not affect basal secretion of ApoA1 in the control medium (BSA) (Figure 4E,G), OA-induced secretion of ApopA1 was significantly reduced in *Ufbp1*^KD^ cells and further decreased by double knockdown of *Ufbp1* and *IRE1α* (Figure 4F,H). Of note, this result appeared different from Oil Red O staining result described in Figure 3B, and this discrepancy may be derived from the different sensitivities and the assays. We also examined subcellular localization of Ufbp1 and ApoB48-GFP. Ufbp1 antibody specificity was validated by staining of Ufbp1 KO cells (Supplemental Fig. S7A). Co-localization of ApoB48-GFP and Ufbp1 at the ER was observed in COS7 cells (Supplemental Fig. S7B), and the interaction between ApoB48 and Ufbp1 was detected in Co-IP assay (Supplemental Fig. S7C). Interestingly, immunostaining also showed that Ufbp1 was co-localized with ApoB48-GFP in C2BBe1 cells, and this co-localization was enhanced by OA treatment (Figure 4I,J). Collectively, these results suggest that Ufbp1 is involved in OA-induced trafficking and secretion of apolipoproteins.

### 3.7 Knockout of intestinal *Ufbp1* in adult mice attenuated high-fat diet-induced hyperlipidemia

**Figure 5:**
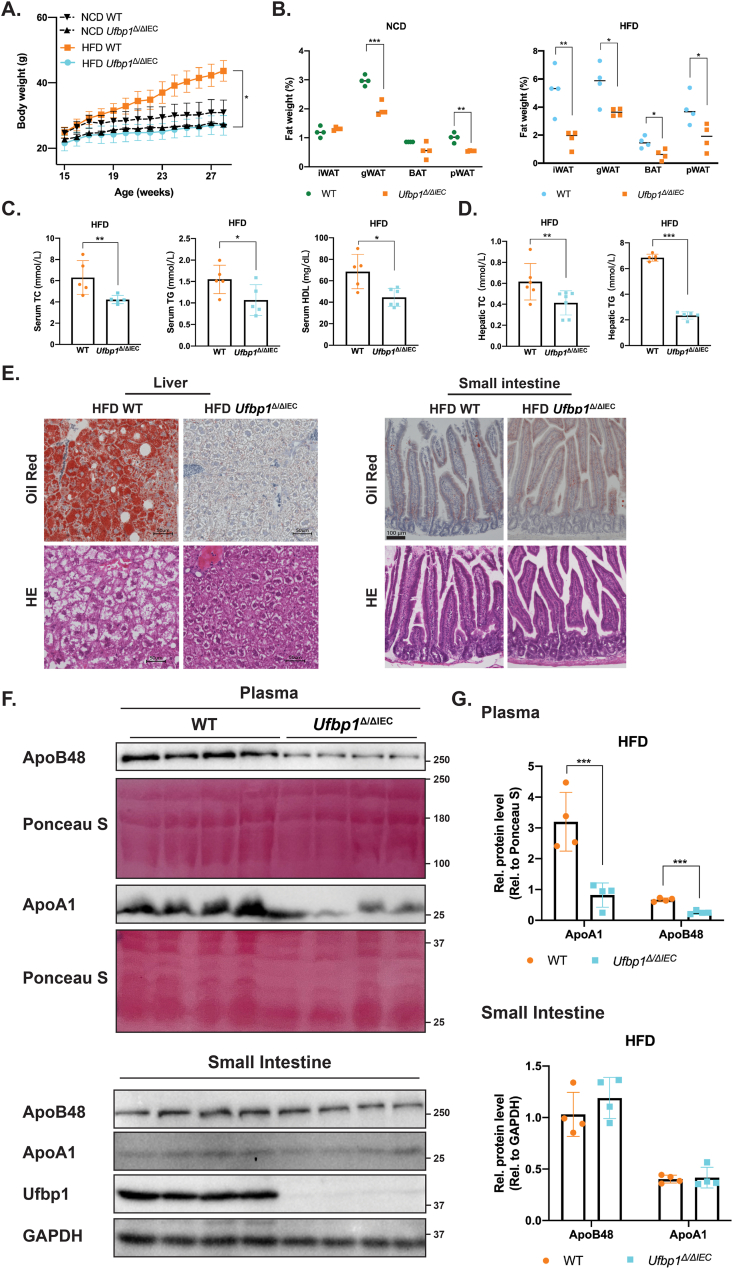
**Deletion of intestinal Ufbp1 attenuated HFD-induced obesity** (A) Growth curves of WT and *Ufbp1*^Δ/ΔIEC^ mice fed on a normal chow diet (NCD) or high-fat diet (HFD). Data were represented as mean ± SEM (n = 4 mice per group). Statistical significance was determined using one-way ANOVA with Tukey's post hoc test. ∗*P* < 0.05. (B) Relative fat weights (normalized to body weight) of male WT or *Ufbp1*^Δ/ΔIEC^ mice on NCD or HFD for 13 weeks (n = 4 mice per group). Statistical significance was determined using Independent-Samples *t* test. ∗*P* < 0.05, ∗∗*P* < 0.01; ∗∗∗*P* < 0.001. (C) Plasma TC, TG, and HDL levels in HFD-fed mice (n = 5 mice per group). (D) Hepatic TC and TG of male WT or *Ufbp1*^Δ/ΔIEC^ mice on HFD for 13 weeks (n = 5 mice per group). Statistical significance was determined using Independent-Samples *t* test. ∗*P* < 0.05; ∗∗*P* < 0.01; ∗∗∗*P* < 0.001. (E) Oil Red O and H&E staining of liver and small intestine tissues of male WT or *Ufbp1*^Δ/ΔIEC^ on HFD for 13 weeks. Scale bar, 50 μm. (F) Immunoblotting of ApoB48 and ApoA1 proteins in the plasma and small intestine tissues of HFD-fed WT and *Ufbp1*^Δ/ΔIEC^ mice. (G) Quantitation of relative levels of ApoB48 and ApoA1 in the plasma and small intestine tissues. Data are represented as mean ± SEM. Statistical significance was determined using Independent-Samples *t* test. ∗∗∗*P* < 0.001 (n = 4).

Although *Ufbp1*^Δ/ΔIEC^ mice did not exhibit any developmental and growth abnormalities, we speculated that adult *Ufbp1*^Δ/ΔIEC^ mice may behave differently in response to metabolic stresses given its involvement in lipid transport at post-natal stage. To investigate the physiological relevance of Ufbp1 in lipid metabolism in adult animals, we subjected *Ufbp1*^Δ/ΔIEC^ mice and littermate controls to either a high-fat diet (HFD) or normal chow diet (NCD) starting at 15 weeks of age. After 13 weeks of feeding, *Ufbp1*^Δ/ΔIEC^ mice exhibited no significant phenotypic alterations under NCD, including body weight (Figure 5A), adiposity (Supplemental Fig. S8A and Figure 5B, except gonad white adipose tissue (gWAT) and perivascular WAT (pWAT)), plasma lipid profiles (Supplemental Fig. S8B, except total cholesterol (TC) and high-density lipoprotein (HDL)), glucose or insulin tolerance (Supplemental Fig. S8D), and hepatic lipid accumulation (Supplemental Fig. S8C). In contrast, HFD-fed *Ufbp1*^Δ/ΔIEC^ mice displayed markedly decreased adipose depot weights compared to WT controls (Supplemental Fig. S8A and Fig.5B). This was accompanied by significantly reduced blood glucose levels in oral glucose tolerance test (OGTT) and insulin tolerance test (ITT) (Supplemental Fig. S8D), along with decreased total cholesterol (TC), triglyceride (TG), and high-density lipoprotein (HDL) in the blood and liver (Figure 5C,D).

Histological analysis revealed no overt differences in the small intestines and livers between WT and *Ufbp1*^Δ/ΔIEC^ mice under normal diet (NCD) (Supplemental Fig. S8E). However, hepatic steatosis was markedly attenuated in HFD-fed *Ufbp1*^Δ/ΔIEC^ mice, as evidenced by HE and Oil Red O staining (Fig.5E). In addition, Oil Red O staining confirmed increased lipid accumulation in the intestinal villi of *Ufbp1*^Δ/ΔIEC^ mice following HFD challenge (Fig.5E).

We further examined the levels of apolipoproteins in both plasma and tissues. Under NCD, plasma ApoB48 was slightly decreases and ApoA1 remained no change even though both intestinal ApoB48 and ApoA1 were increased (Supplemental Fig. S8F–I). Interestingly, plasma levels of ApoB48 and ApoA1 in HFD-fed *Ufbp1*^Δ/ΔIEC^ mice were significantly decreased, while their levels in intestinal villi remained comparable to the ones of WT mice (Figure 5F,G). Together, these results strongly suggest the critical role of Ufbp1 in lipid metabolism under high-fat stress.

### 3.8 Knockdown of the UFMylation pathway disrupted secretion of lipoproteins

**Figure 6:**
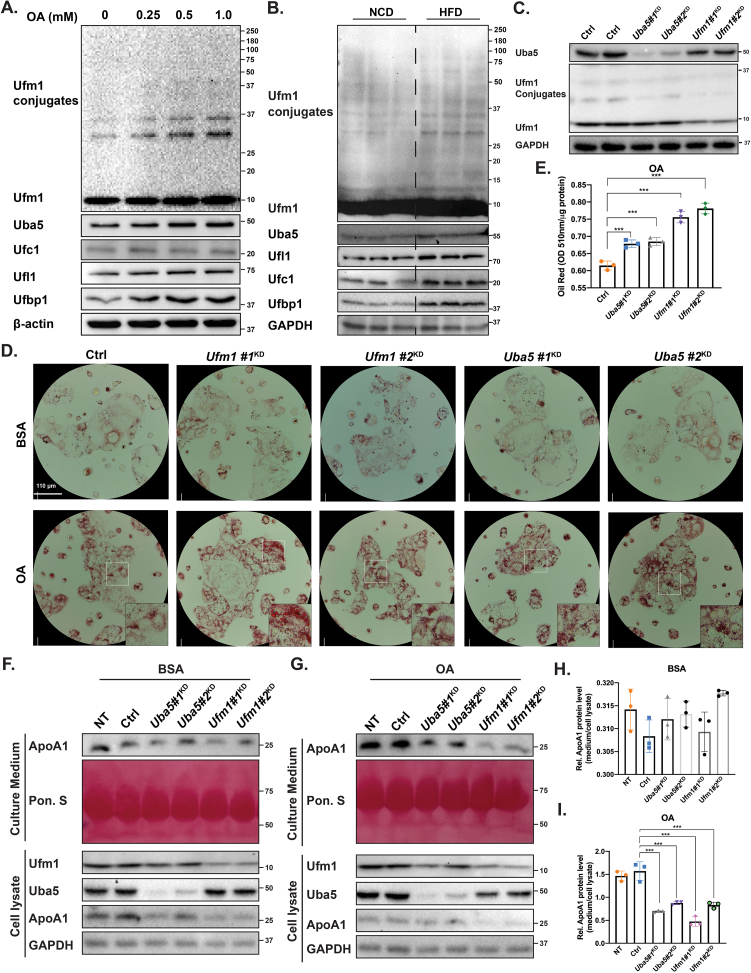
**Knockdown of the UFMylation pathway disrupted secretion of lipoproteins** (A) Elevated protein levels of Ufm1, Uba5, Ufc1, Ufl1, Ufbp1 and Ufm1 conjugates in OA-treated C2Bbe1 cells. β-actin serves as a loading control. (B) HFD-induced increase of UFMylation proteins in the small intestine of mice fed with HFD. Mice were fed with HFD for 13 weeks, and cell lysates of small intestine were subjected to immunoblotting analysis. (C) Representative Western blot images showing protein levels of Ufba5 and Ufm1 in control (vector), *Uba5*^KD^, and *Ufm1*^KD^ C2Bbe1 cells. GAPDH serves as a loading control. (D) Representative images of Oil Red O staining in control (vector), *Uba5*^KD^, and *Ufm1*^KD^ C2Bbe1 cells after 8 h of 0.5 mM oleic acid (OA) treatment. Lipid droplets are stained red. Scale bar, 110 μm. (E) Quantification of Oil Red O intensity. Dimethyl sulfoxide (DMSO) was added to each sample; after shaking at room temperature for 5 min, the density of samples were read at 510 nm on a spectrophotometer. Data represent mean ± SEM (n = 3), analyzed by one-way ANOVA with Tukey's post hoc test. ∗∗∗*P* < 0.001. (F) and (G) ApoA1 levels in culture media and cell lysates in BSA and OA-treated C2Bbe1 cells. Control and knockdown cells were incubated with DMEM containing either BSA or 0.5 mM OA complexed with BSA. Media were collected after an overnight incubation. Representative Western blot images showing protein levels of ApoA1, Uba5 and Ufm1 in non-treatment (NT), control (vector), *Uba5*^KD^, and *Ufm1*^KD^ C2Bbe1 cells. ApoA1 was measured in the cell lysate and media. Ponceau S and GAPDH serves as a loading control. (H) and (I) Quantitation of relative ApoA1 levels in culture media and cell lysates. Data are presented as mean ± SEM (n = 3). Statistical significance was determined using one-way ANOVA with Tukey's post hoc test. ∗∗∗*P* < 0.001.

Ufbp1 is the co-factor of Ufm1-speciifc E3 ligase Ufl1, and its deficiency disrupts UFMylation of its principal target RPL26 [29,30]. Therefore, we attempted to determine whether UFMylation is also involved in lipid metabolism. As shown in Figure 6A, OA treatment induced a substantial increase of UFMylation components and Ufm1 conjugation (Fig.6A), while HFD also led to the elevation of UFMylation proteins in the small intestine (Fig.6B). These results suggest that UFMylation is dynamically regulated in response to lipid exposure. To assess the functional relevance of UFMylation in lipid homeostasis, we knockdowned either Uba5 (E1 enzyme) or Ufm1 in C2BBe1 cells and then examined LD levels in OA-treated cells. Oil Red O staining demonstrated marked lipid accumulation in *Uba5*^KD^ or *Ufm1*^KD^ cells compared to control cells (Figure 6C–E), indicating that deficiency of the UFMylation pathway causes intracellular lipid deposition. We also examined lipoproteins in these knockdown cells. Although either *Uba5* or *Ufm1* knockdown appeared to affect the intracellular level of ApoA1 protein (Fig.6F), OA-treated conditioned media showed a marked reduction (relative to intracellular level) in secreted ApoA1 from *Uba5*^KD^ or *Ufm1*^KD^ cells (Figure 6F–I), indicating impaired lipoprotein secretion. This result suggests that the UFMylation pathway is indeed critical for lipoprotein secretion and lipid trafficking.

### 3.9 UFMylation regulated the recruitment of SarI1B to ERES of ApoB48 protein

**Figure 7:**
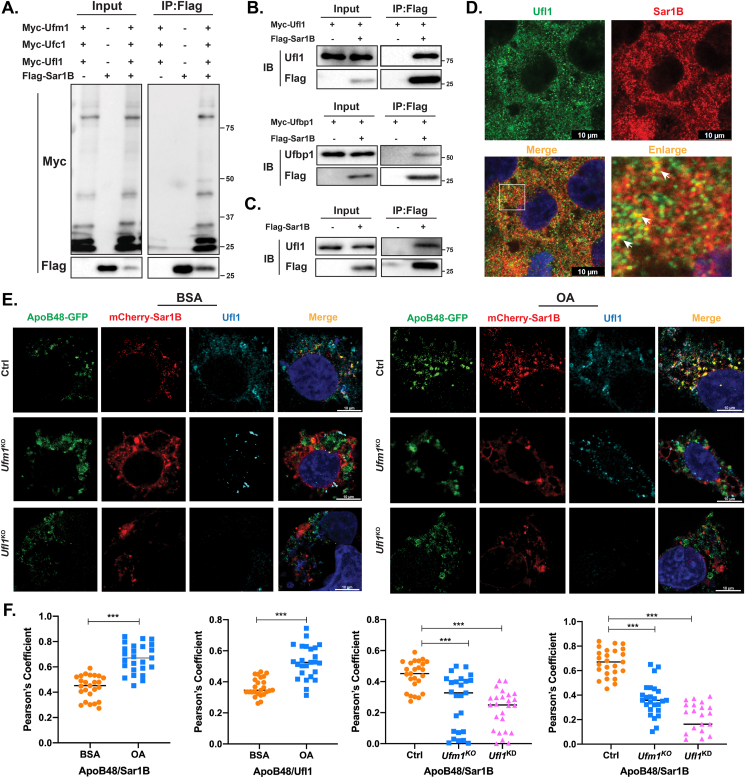
**The UFMylation pathway interacted with COPII complex to regulate lipoprotein secretion** (A) Co-IP of Flag-Sar1B and Ufm1 conjugates. *Ufsp2*^KO^ HEK293T cells were transiently co-transfected with Ufm1-Myc, Ufc1-Myc, Ufl1-Myc, and flag-Sar1B. Cell lysates were immunoprecipitated with anti-Flag antibody and analyzed by immunoblotting using the indicated antibodies. (B) Co-IP assays of Myc-ufl1, Myc-Ufbp1 and Flag-Sar1B. HEK293T cells were transiently co-transfected with Myc-ufl1, Myc-Ufbp1 and Flag-Sar1B. Cell lysates were immunoprecipitated with anti-Flag antibody and analyzed by immunoblotting using the indicated antibodies. (C) Co-IP assays of endogenous Ufl1 and Flag-Sar1B. HEK293T cells were transiently co-transfected with Flag-Sar1B. Cell lysates were immunoprecipitated with anti-Ufl1 antibody and analyzed by immunoblotting using the indicated antibodies. (D) Co-localization of endogenous Sar1B and Ufl1 in C2BBe1 cells. Cells was immunofluorescence (IF) stained with Sar1B and Ufl1 antibodies. Images were captured using Leica Stellaris 5 confocal microscope with 63x lens. Scale bar, 10 μm. (E) Co-localization of Sar1B, ApoB48, and Ufl1 in WT and UFMylation knockdown COS7 cells. COS7-MTP cells (WT, Ufm1^KO^ and Ufl1^KD^) were transiently transfected with ApoB48-GFP and mCherry-Sar1B constructs and then treated with BSA or OA as indicated. Confocal images were acquired using Leica Stellaris 5 confocal microscope with 63x lens. (F) Colocalization was quantified using Pearson's correlation coefficient. Date are presented as mean ± SEM (n = 25 cells). Statistical significance was determined using one-way ANOVA with Tukey's post hoc test. ∗∗∗*P* < 0.001.

We next investigated the underlying mechanism of UFMylation's involvement in lipoprotein secretion. Given the essential role of COPII-mediated ER export in lipoprotein trafficking, we examined potential interaction between the components of the COPII machinery and UFMylation pathway. Co-immunoprecipitation (co-IP) assays revealed that GFP-Sar1B, Sec23A, Sec23B and Sec16L, but not GFP-Sec24C and Sec24, were present in Flag-Ufm1 immunoprecipitate (Supplemental Fig. S9A), indicating a potential unrecognized link between the UFMylation system and the COPII machinery. Additionally, Flag-tagged Sar1B pulled down various Ufm1 conjugates in *Ufsp2*^KO^ HEK293T cells in which Ufm1 conjugates are enriched, suggesting that Sar1B may interact with UFMylated proteins (Figure 7A). Interestingly, Flag-Sar1B interacted well with both Myc-Ufl1 and Myc-Ufbp1, two components of Ufm1 E3 ligase complex (Fig.7B). It appeared that Flag-Sar1B had a higher affinity to Myc-Ufl1 than Myc-Ufbp1 (Figure 7B, comparing the relative levels of immunoprecipitated and input Myc-tagged proteins). Endogenous Ufl1 but not Ufbp1 was pulled down by Flag-Sar1B (Fig.7C). Moreover, exogenous GFP-Ufl1 and mCherry-Sar1B were partially co-localized in U2OS cells (Supplemental Fig. S9B). Finally, endogenous Ufl1 and Sar1B were partially co-localized in both U2OS cells (Supplemental Fig. S9C) and C2BBe1 cells (Fig.7D). Together, these results suggest a potential direct interaction between the COPII coat complex and the UFMylation components.

Sar1B is a critical small GTPase that initiates the assembly of COPII coat at ER exit site of ER-located cargo proteins, and its mutations cause Chylomicron Retention Disease (CRD) [31]. Given the interaction between Ufl1 and Sar1B, we next examined whether UFMylation affected the recruitment of COPII coat to the ERES of lipoproteins. In COS7 cells, OA treatment markedly enhanced the co-localization of exogenous GFP-ApoB48 and mCherry-Sar1B comparing to BSA control (Figure 7E,F). Interestingly, OA treatment also increased the co-localization of GFP-ApoB48 with endogenous Ufl1, indicating that lipid overloading may also promotes the recruitment of Ufl1 to ER-located lipoprotein (Figure 7E,F). Importantly, disruption of the UFMylation pathway by knockdown of either Ufm1 or Ufl1 significantly reduced the co-localization between ApoB48 and Sar1B (Figure 7E,F). Taken together, our results demonstrate that the UFMylation pathway is a critical player to regulate the initial step of CM assembly in the enterocytes during cellular response to lipid stress.

## 4 Discussion

In this study, we reported a serendipitous finding of a novel function of the UFMylation pathway in lipid metabolism in intestinal enterocytes. Previous studies have shown that intestine-specific deletion of *Ufbp1*, a key component of the UFMylation system, causes elevation of ER stress and UPR activation and leads to remarkable loss of professional exocrine secretory cells such as Paneth and goblet cells in the intestine [17], yet no significant functional defect or abnormality has been found in *Ufbp1*^Δ/ΔIEC^ enterocytes. Inactivation of either PERK or IRE1α failed to prevent the loss of exocrine cells in *Ufbp1*^Δ/ΔIEC^ intestine (Supplemental Figs. 1 and 2), suggesting that other signaling pathways including the ATF6 branch may be involved in loss of *Ufbp1* deficient cells. Surprisingly, simultaneous deletion of *Ufbp1* and *IRE1α* led to dramatic buildup of lipid droplets in the enterocytes during post-natal development (Fig.1), strongly suggesting that Ufbp1 is also important for enterocyte function in lipid metabolism. To validate our initial findings, we further tested adult *Ufbp1*^Δ/ΔIEC^ mice in response to lipid stress and found that they were highly resistant to high-fat diet-induced hyperlipidemia (Fig.5). Furthermore, we used cell models to demonstrate that knockdown of *Ufbp1* and other UFMylation components impaired oleic acid-induced secretion of apolipoproteins and led to accumulation of lipid droplets (Figure 3, Figure 6). Collectively, our results strongly suggest that the UFMylation pathway including Ufbp1 protein plays a pivotal role in lipid transport in enterocytes.

Assembly of pre-CMs in the ER is an initiating event for lipid transport in enterocytes [6,7]. It starts with co-translational lipidation of ApoB48 with the help of MTP and protein disulfate isomerase (PDI) [32,33]. Intestine-specific deletion of murine *Mtp* gene results in a significant reduction of plasma ApoB48 protein [34]. More recently, several proteins have been shown to be involved in the initial lipidation and assembly of CMs, including Transmembrane 6 superfamily member 2 (TM6SF2) [35], Surfeit locus protein 4 (SURF4) [[36], [37], [38]], Proline-rich acidic protein 1 (PRAP1) [39], Phospholipase A2 group B12 (PLA1G12B) [40], and Cell death-inducing DFFA-like effector b and c (CIDEB and CIDEC) [41,42]. Loss of these proteins in either cells or animal models leads to impaired CM production, reduced circulating lipids and lipoproteins, and intestinal lipid accumulation, underscoring their critical roles in intestinal lipid metabolism and transport. After lipidation and formation of CMs, they are transported from the ER to the Golgi apparatus where they mature and are eventually secreted via exocytosis. Transport of CMs is dependent on coat protein complex II (COPII) that is essential for cargo selection and ER-to-Golgi trafficking [43]. The components of COPII coat including Sar1B, Sec13/31 and Sec23/24 are found to be present on pre-CM vesicles [44]. Mutations in Sar1B causes chylomicron retention disease [31,45,46]. COPII components Sec23 and Sec24C are required for CM's docking at the cis-Golgi [44]. Our results also showed that depletion of Sec24D in C2BBe1 cells led to intracellular lipid accumulation (Fig.3). In addition, proteins such SURF4 and TANGO1-like protein (TAL1) are also implicated in lipid cargo loading and ER-to-Golgi trafficking [38,47]. Yet the precise interplay between COPII and these accessory factors in intestinal chylomicron secretion remains to be fully elucidated.

**Figure 8:**
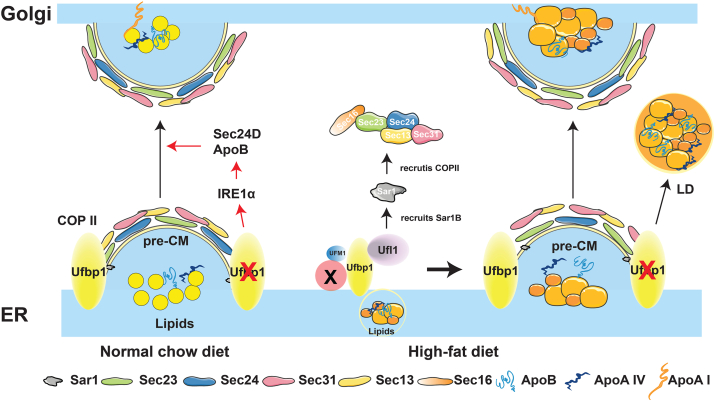
**A working model of UFMylation's role in lipid transport in enterocytes**. Under normal chow diet (NCD) condition, UFMylation plays a modest role in lipid transport in enterocytes. Furthermore, UFMylation deficiency or *Ufbp1* deletion activates the IRE1α pathway, leading to upregulation of ApoB and COPII complex, thereby compensating for impaired lipid handling and maintaining lipid transport. Under high-fat diet (HFD) condition, elevated UFMylation, especially Ufl1 protein, enhances the recruitment of Sar1B and other COPII components to ERES via the Ufl1-Sar1B interaction and promotes the formation of pre-CM complex. Impairment of UFMylation or loss of its components such as Ufbp1 may attenuate lipid export from ER, resulting in intracellular lipid accumulation.

The UFMylation pathway is a newly identified ubiquitin-like modification system [8]. Like other ubiquitin-like systems, it includes E1 enzyme (Uba5), E2 enzyme (Ufc1), and Ufm1-specific E3 ligase (Ufl1 and Ufbp1 complex). Ufbp1 is an ER protein with a N-terminal transmembrane domain that anchors Ufl1/Ufbp1 complex on the ER membrane [11]. In this study, we have provided strong genetic evidence supporting the role of UFMylation in lipid trafficking in intestinal enterocytes. We propose that the UFMylation pathway is critical for high-capacity COPII-mediated lipid transport in enterocytes (Figure 8). In adult mice fed with NCD, the UFMylation pathway plays a modest role in CM transport that is mainly handled by COPII-mediated trafficking. Furthermore, *Ufbp1* deletion is compensated by IRE1α-mediated up-regulation of Sec24D and other COPII components (Figure 2, Figure 3). Therefore, *Ufbp1* knockout had a modest impact on lipid homeostasis under normal lipid load. Nonetheless, Ufbp1 may still affect systemic lipid levels, as indicated by less gWAT and pWAT tissues and lower plasma TC and HDL in *Ufbp1*^Δ/ΔIEC^ mice fed with NCD (Supplemental Fig. S7 and Fig.5). In contrast, under high lipid load, the UFMylation pathway including Ufbp1 is required for high capacity COPII-mediated lipid transport, and *Ufbp1* deficiency leads to LD accumulation in enterocytes and resistance to HFD-induced hyperlipidemia. We found that Sar1B interacted well with Ufl1/Ufbp1 complex and other UFM1 conjugates (Figure 7 and Supplemental Fig. S8), strongly indicating a direct link between UFMylation components and COPII complex. Importantly, knockdown of the UFMylation pathway caused reduced recruitment of Sar1B to ApoB48, suggesting that UFMylation regulates the early step of CM assembly. Based upon our results, we propose that in response to lipid overload, accumulation of lipids and lipoproteins in the ER lumen leads to elevated UFMylation of RPL26 or other unknown target proteins and local enrichment of Ufl1/Ufbp1 complex, which in turn recruits Sar1B and other COPII components to the ER exit site and promotes pre-CM assembly (Fig.8).

During post-natal development, mouse milk lipid content is typically around 20–40% of the total milk composition and may be considered as a diet with relatively high fat. The question is why *Ufbp1*^Δ/ΔIEC^ pups exhibit normal development without LD accumulation in the enterocytes. One explanation is that IRE1α-mediated up-regulation lipid transport machinery in *Ufbp1*^Δ/ΔIEC^ pup may contribute to its increasing capacity of handling lipid transport. Knockout of *IRE1α* blocks the up-regulation of Sec24D and other components induced by *Ufbp1* deletion and lowers lipid transport capacity, thereby causing remarkable accumulation of LDs in the enterocytes of *Ufbp1*;*IRE1α DKO*^IEC^ mice. Additionally, our RNA-seq result also showed that other cellular processes were affected in these KO tissues. The genes were associated with vacuole, lysosome, and lipid metabolism were significantly enriched in *Ufbp1*^Δ/ΔIEC^ tissues (Fig.2A), indicating that elevation of catabolic and degradative pathways may also contribute to lipid handling capacity of *Ufbp1* deficient enterocytes. Ribosomal biogenesis was enhanced in *Ufbp1*;*IRE1α DKO*^IEC^ tissues (Fig.2A), a phenomenon that was also observed in other UFMylation deficient intestinal tissues [48]. Given the fact that RPL26 is the principal UFM1 target [29,30], it is very likely that regulation of ribosomal biogenesis is an important function of UFMylation. Yet it remains to be investigated if the change of protein translational capacity is related to altered lipid metabolism in the enterocytes. Finally, either immature or mature enterocytes in post-natal mice may handle lipid absorption and transport differently from the ones in adult mice. More studies will be needed to further explore the function and mechanism of UFMylation in other cellular processes and its relationship to lipid metabolism in the enterocytes.

There are some limitations in our study. For example, chow diet and high-fat diet differ not only in fat content but also in other nutritional components, and therefore, may introduce additional metabolic variables. Nonetheless, our current study of both *in vivo* and *in vitro* models has presented strong evidence for the involvement of the UFMylation pathway in regulation of lipid transport. Apparently, many questions remain regarding the underlying mechanism. Does UFMylation affect other lipid metabolic processes in enterocytes? How does lipid overload promote UFMylation? How does Ufl1 interact with Sar1B, and what is the functional impact of this interaction? Is UFMylation involved in other vesicle trafficking processes? More studies would be warranted to further test our proposed model and answer these important questions. Intestinal enterocytes process dietary lipids and transport them throughout the body, and improper lipid handling is linked to health problems like obesity and cardiovascular diseases [1]. Therefore, lipid absorption and transport in the enterocytes would be legitimate target of pharmacological intervention for treatment of these diseases. Our study has demonstrated a pivotal role of UFMylation in lipid metabolism in the intestine, which may provide a novel intervention target for combating health problems associated with lipid metabolism.

## CRediT authorship contribution statement

**Yaqun Wang:** Writing – original draft, Visualization, Validation, Methodology, Investigation, Formal analysis, Data curation. **Feng Zhou:** Validation, Methodology, Investigation, Formal analysis, Data curation. **Xin Xu:** Validation, Resources, Methodology, Investigation, Formal analysis, Data curation. **Guangyu Wu:** Resources, Methodology, Investigation, Formal analysis, Conceptualization. **Hong Xu:** Writing – original draft, Validation, Supervision, Project administration, Methodology, Investigation, Funding acquisition, Formal analysis, Data curation, Conceptualization. **Honglin Li:** Writing – original draft, Validation, Supervision, Project administration, Investigation, Funding acquisition, Formal analysis, Conceptualization.

## Grant

The authors gratefully acknowledge the financial support from National Institute of Health (1R01DK113409) to HL, the National Science Foundation of China (No. 82260639) and the “Double Thousand Plan” Science and Technology Innovation High-end Talent project of Jiangxi Province (No. Jxsq2023201032).

## Declaration of competing interest

The authors claim no competing interest.

## Data Availability

Data will be made available on request.
