# Supplementary material for: A UFMylation-COPII axis orchestrates lipid transport in intestinal enterocytes and regulates systemic lipid balance
Source: Mol Metab. 2026 Jun 12;110:102397. doi: 10.1016/j.molmet.2026.102397 (PMC13320283; doi:10.1016/j.molmet.2026.102397)
Supplement: Multimedia component 1 [file mmc1.pdf]

Supplemental Materials:

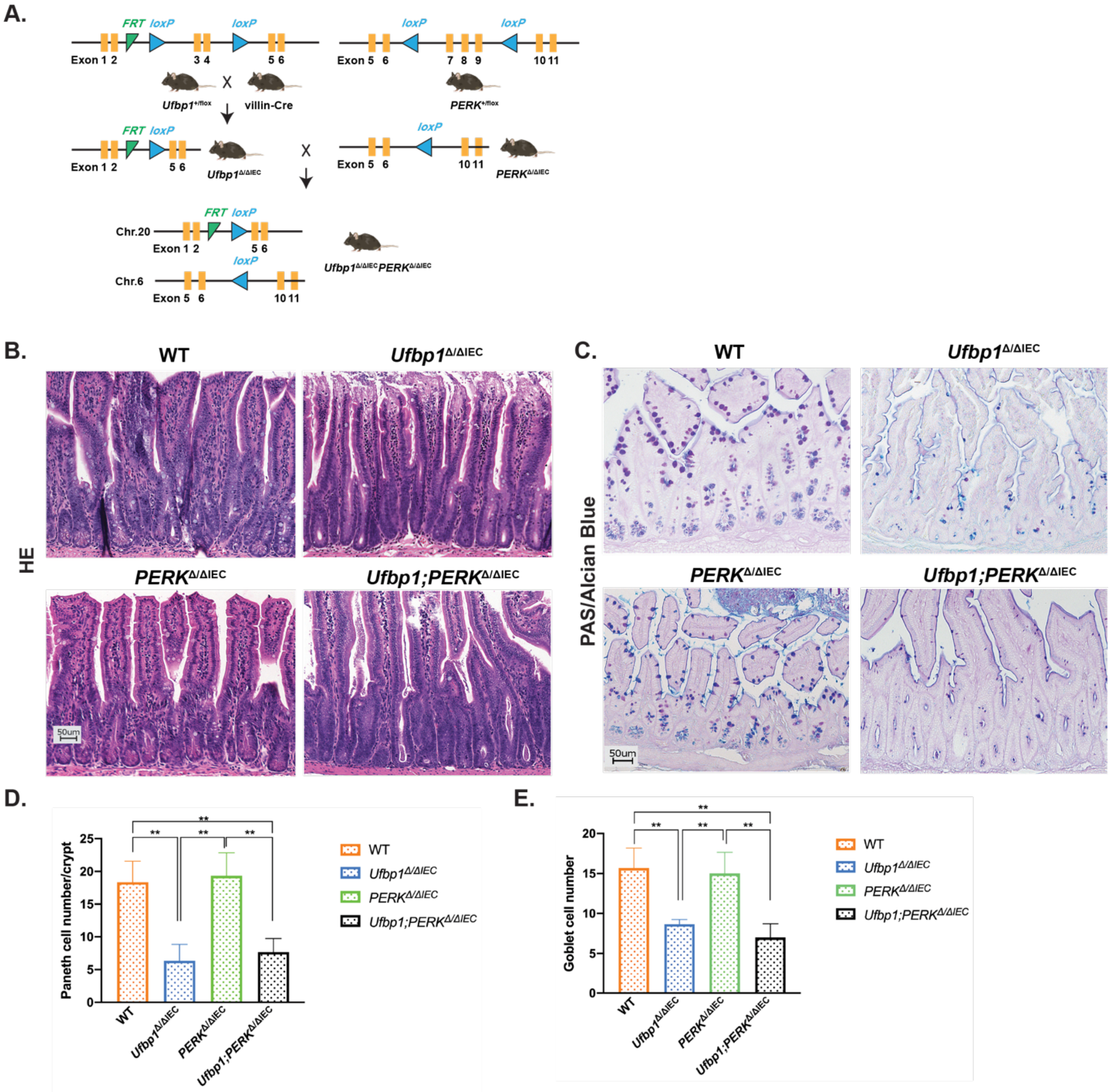

Figure S1. (A) Generation of *Ufbp1* and *PERK* conditional knockout (CKO) mice. The CKO mice were obtained by crossing *Ufbp1* $^{f/f}$ and *PERK*$^{f/f}$ mice with Villin-Cre transgenic mice, which specifically express Cre recombinase in intestinal epithelial cells (IECs). *DKO*^{IEC} mice (*Ufbp1*$^{\Delta/\Delta IEC}$;*PERK*$^{\Delta/\Delta IEC}$) were generated by breeding *Ufbp1* $^{f/f}$*; PERK*$^{f/f}$ mice with Villin-Cre mice. (B and C) Representative H&E and PAS/Alcian Blue-stained sections of WT, *Ufbp1*$^{\Delta/\Delta IEC}$, *PERK*$^{\Delta/\Delta IEC}$, and *Ufbp1*$^{\Delta/\Delta IEC}$*;PERK*$^{\Delta/\Delta IEC}$ intestine tissues. Scale bar, 50 μm. (D and E) quantitation of Paneth and goblet cells in WT, *Ufbp1*$^{\Delta/\Delta IEC}$, *PERK*$^{\Delta/\Delta IEC}$, and *Ufbp1*$^{\Delta/\Delta IEC}$*;PERK*$^{\Delta/\Delta IEC}$ intestine. At least 100 crypts and villi were counted from 6 mice of each genotype in a double blinded manner. The average numbers of Paneth and goblet cells per crypt/villus axis were scored. Data are represented as mean ± SEM. Statistical significance was determined using one-way ANOVA with Tukey's post hoc test. ***P* < 0.01.

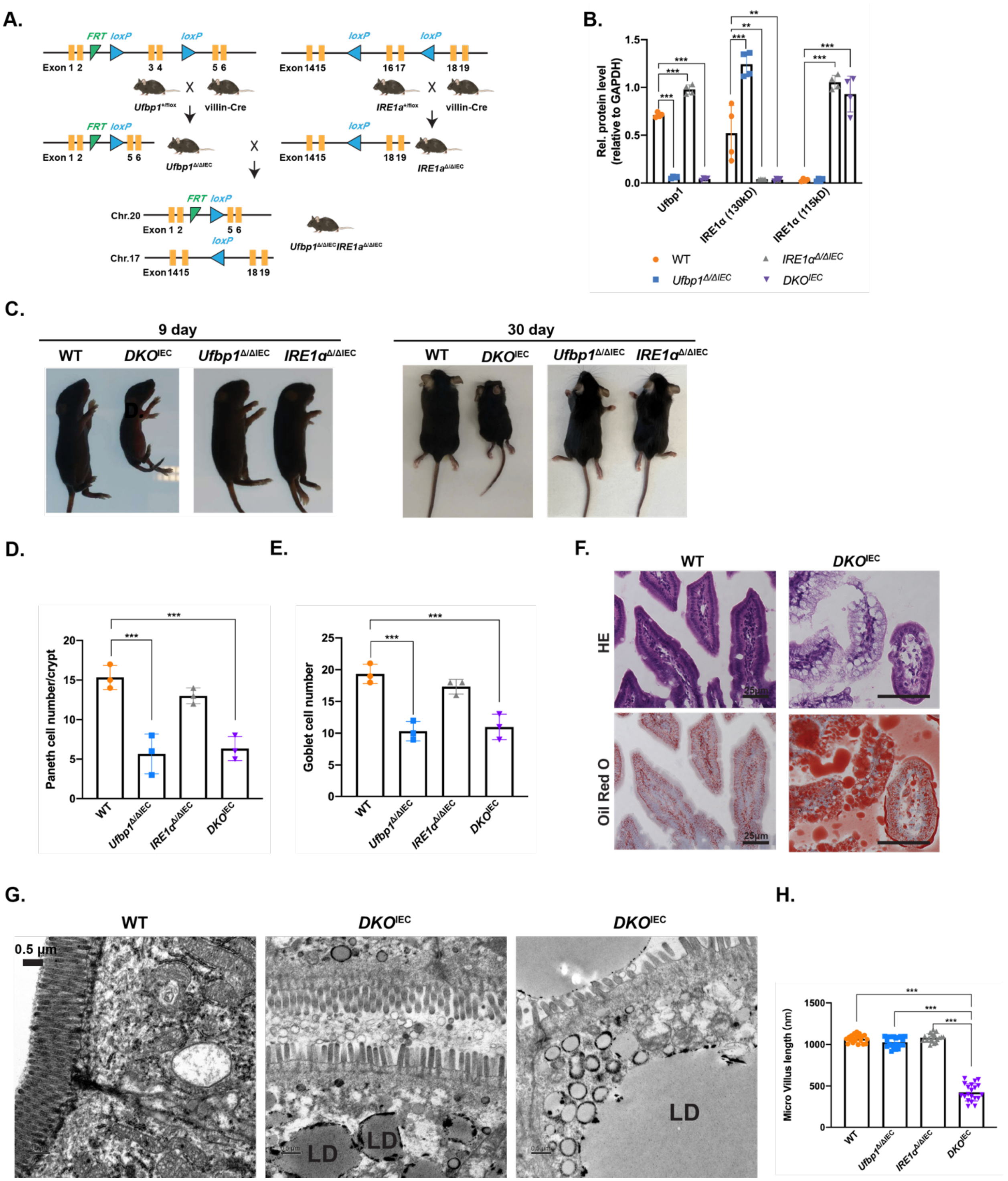

A.
FRT loxP
loxP
Exon 1 2
3 4
5 6
Ufbp1+/flox
villin-Cre
Exon 1415
1617
1819
IRE1a+/flox
Ufbp1Δ/ΔIEC
IRE1aΔ/ΔIEC
Chr.20
Chr.17
Ufbp1Δ/ΔIECIRE1aΔ/ΔIEC
B.
Rel. protein level (relative to GAPDH)
Ufbp1
IRE1α (130kD)
IRE1α (115kD)
WT
Ufbp1Δ/ΔIEC
IRE1αΔ/ΔIEC
DKOIEC
C.
9 day
30 day
WT
DKOIEC
Ufbp1Δ/ΔIEC
IRE1aΔ/ΔIEC
D.
Paneth cell number/crypt
E.
Goblet cell number
F.
WT
DKOIEC
HE
Oil Red O
25μm
G.
WT
DKOIEC
0.5 μm
LD
H.
Micro Villus length (nm)

Figure S2. (A) Generation of *Ufbp1* and *IRE1α* conditional knockout (CKO) mice. The CKO mice were obtained by crossing *Ufbp1* $^{f/f}$ and *IRE1α*$^{f/f}$ mice with Villin-Cre transgenic mice, which specifically express Cre recombinase in intestinal epithelial cells (IECs). *DKO*^{IEC} mice (*Ufbp1*$^{\Delta/\Delta IEC}$; *IRE1α* $^{\Delta/\Delta IEC}$) were generated by breeding *Ufbp1*$^{f/f}$*IRE1α*$^{f/f}$ mice with Villin-Cre mice. (B) Quantification of Ufbp1 and IRE1a protein levels normalized to GAPDH. Data are presented as mean ± SEM (n = 4). Statistical significance was determined using one-way ANOVA with Tukey's post hoc test. $^{**}P < 0.01$; $^{***}P < 0.001$. (C) Representative images of WT, *Ufbp1*$^{\Delta/\Delta IEC}$, *IRE1α*$^{\Delta/\Delta IEC}$, and *DKO*^{IEC} mice at post-natal 9-day and 30-day, highlighting severe growth retardation in *DKO*^{IEC} mice. (D and E) Quantitation of Paneth and goblet cells in the intestines of WT or *Ufbp1*$^{\Delta/\Delta IEC}$ or *IRE1α*$^{\Delta/\Delta IEC}$ or *DKO*^{IEC} mice at post-natal day 30. At least 100 crypts were counted from 3 mice of each genotype in a double blinded manner. Data are represented as mean ± SEM. The average numbers of Paneth and goblet cells per crypt and villus were scored. Statistical significance was determined using one-way ANOVA with Tukey's post hoc test. $^{***}P < 0.001$. (F) H&E staining of small intestinal sections from WT and *DKO*^{IEC} mice revealed the presence of vacuole-like structures in the intestinal epithelium of *DKO*^{IEC} mice. To determine whether these structures represent lipid accumulation, Oil Red O staining was performed on adjacent tissue sections. Comparative analysis demonstrated that the vacuoles observed in H&E staining corresponded to lipid deposits, as indicated by Oil Red O-positive staining in *DKO*^{IEC} mice but not in WT controls. Scale bars, 25 μm. (G) High-magnification TEM images of micro-villi of WT, *Ufbp1*$^{\Delta/\Delta IEC}$, *IRE1α* $^{\Delta/\Delta IEC}$ and *DKO*^{IEC} enterocytes. (H) Quantitation of micro-villi length. Data are represented as mean ± SEM. Statistical significance was determined using one-way ANOVA with Tukey's post hoc test. $^{***}P < 0.001$ (n = 20).

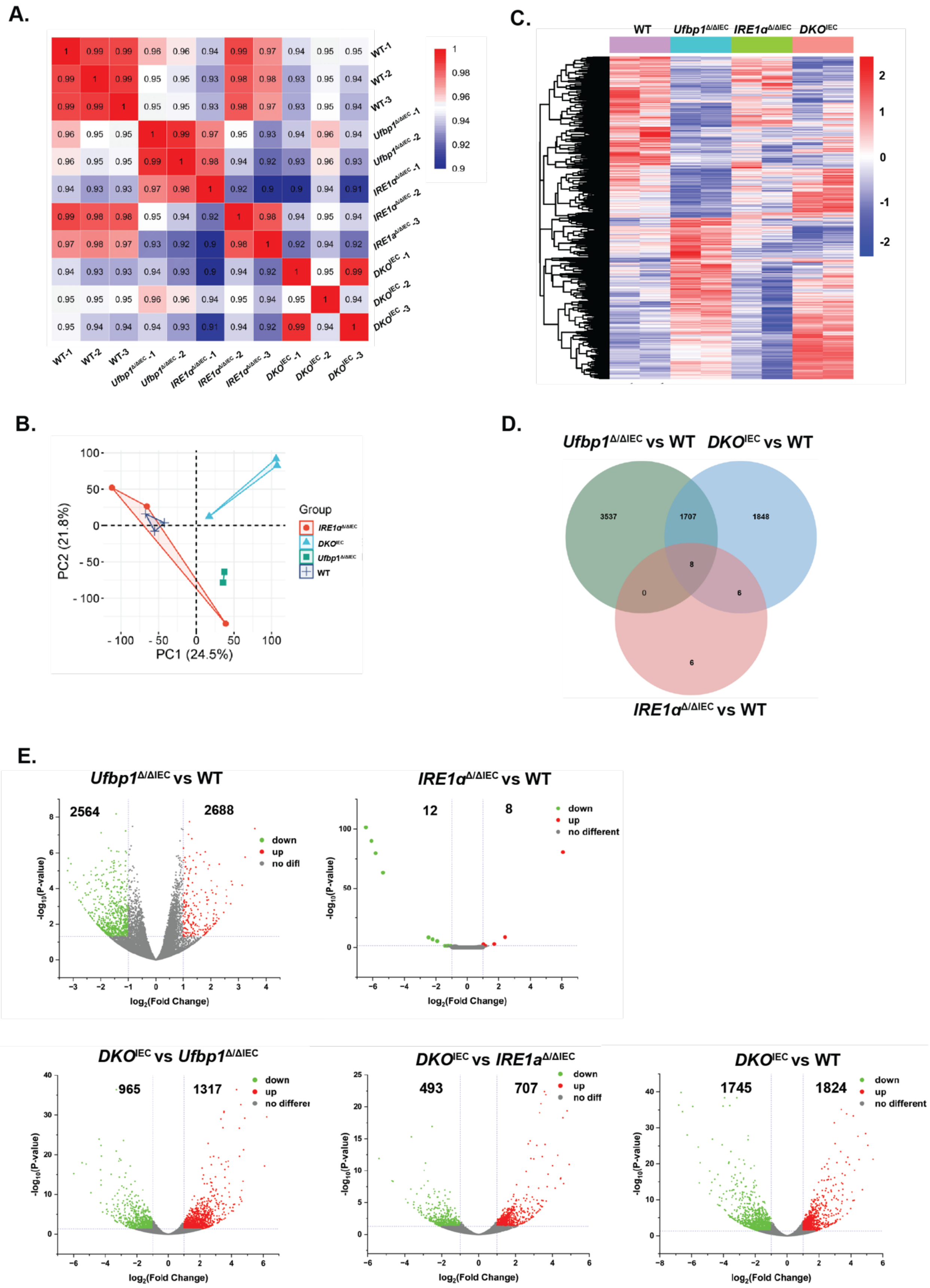

A.
C.
WT
Ufbp1ΔIEC
IRE1αΔIEC
DKOIEC
B.
PC1 (24.5%)
PC2 (21.8%)
Group
D.
Ufbp1ΔIEC vs WT
DKOIEC vs WT
IRE1αΔIEC vs WT
3537
1707
1848
8
0
6
6
E.
Ufbp1ΔIEC vs WT
2564
2688
IRE1αΔIEC vs WT
12
8
DKOIEC vs Ufbp1ΔIEC
965
1317
DKOIEC vs IRE1αΔIEC
493
707
DKOIEC vs WT
1745
1824
down
up
no different
log2(Fold Change)
-log10(P-value)

Figure S3. (A) Pearson correlation heatmap displayed the pairwise correlation coefficients between all samples, confirming high reproducibility and consistency within biological replicates. (B) PCA plot illustrated the global transcriptomic differences between experimental groups. The first two principal components (PC1 and PC2) accounted for the majority of variance. (C) A heatmap showed the expression patterns of DEGs among the four experimental groups. Hierarchical clustering revealed distinct transcriptional signatures, with upregulated (red) and downregulated (blue) genes clearly separated between groups. (D) Venn diagrams illustrated the overlap and specificity of DEGs among the four experimental groups. The numbers of unique and shared genes were indicated for each comparison, highlighting both group-specific transcriptional alterations and commonly dysregulated genes. The intersecting regions represented core DEGs that are consistently altered. (E) Volcano plots provided a global overview of transcriptional changes between experimental groups, with significantly upregulated (red) and downregulated (blue) genes highlighted based on log2(fold change) and -log10(p-value). The threshold for significance was set at $|\log2FC| > 1$ and adjusted $p < 0.05$ (FDR correction).

A.

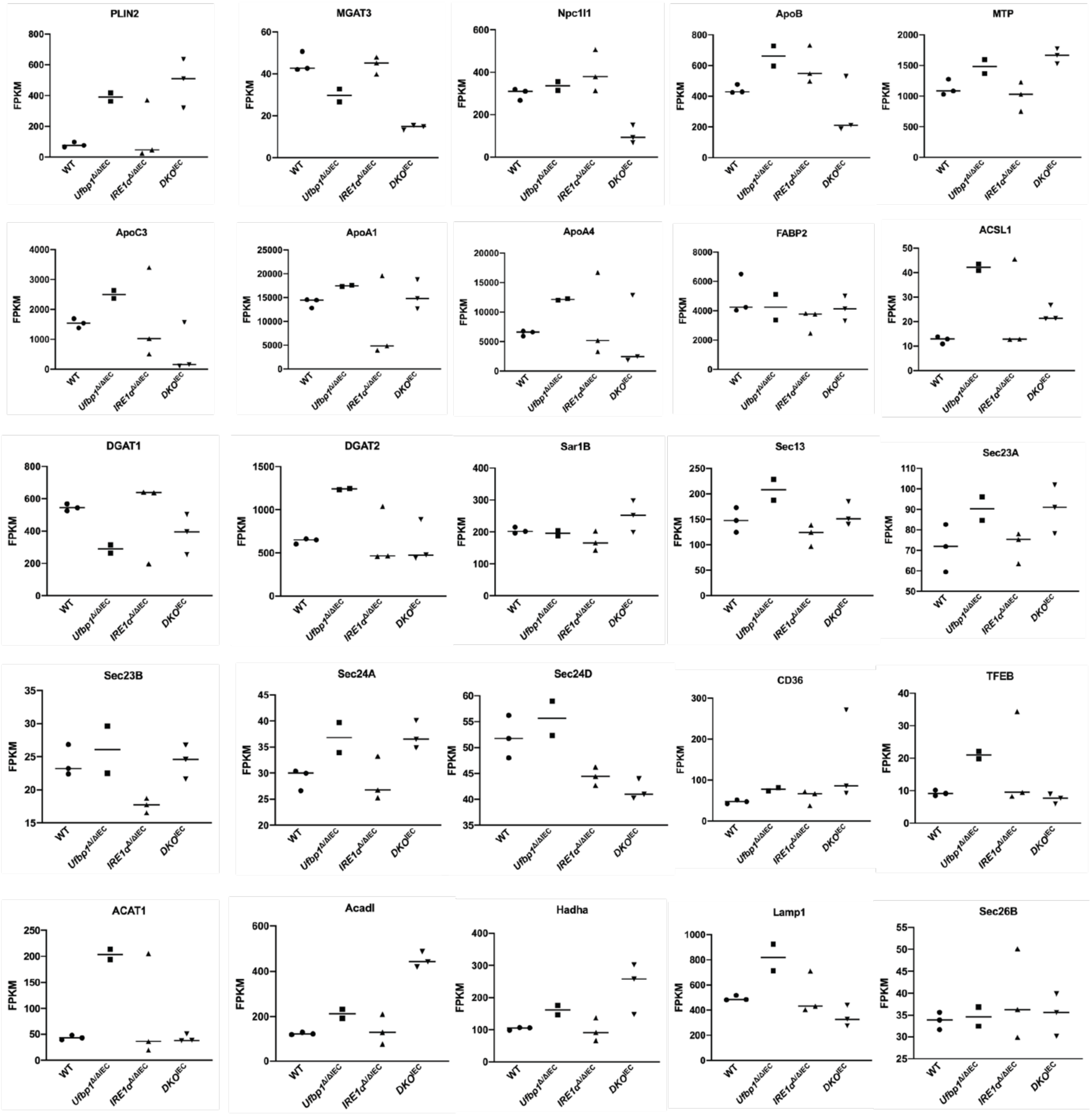
PLIN2
MGAT3
Npc1l1
ApoB
MTP
ApoC3
ApoA1
ApoA4
FABP2
ACSL1
DGAT1
DGAT2
Sar1B
Sec13
Sec23A
Sec23B
Sec24A
Sec24D
CD36
TFEB
ACAT1
Acadl
Hadha
Lamp1
Sec26B
FPKM
WT
Ufbp1Δ/ΔIEC
IRE1αΔ/ΔIEC
DKOIEC

B.

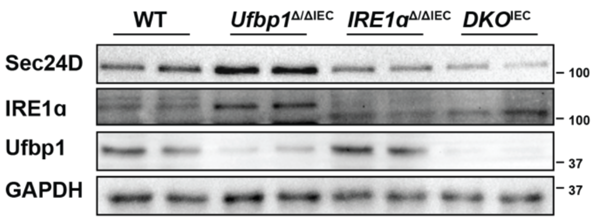
WT
Ufbp1Δ/ΔIEC
IRE1αΔ/ΔIEC
DKOIEC
Sec24D
IRE1α
Ufbp1
GAPDH
100
100
37
37

C.

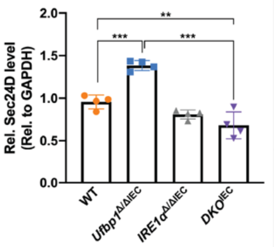
Rel. Sec24D level
(Rel. to GAPDH)
2.0
1.5
1.0
0.5
0.0
**
***
***
WT
Ufbp1Δ/ΔIEC
IRE1αΔ/ΔIEC
DKOIEC

Figure S4. (A) FPKMs of lipid associated genes of DEGs in WT, *Ufbp1*$^{\Delta/\Delta IEC}$, *IRE1α*$^{\Delta/\Delta IEC}$, and *DKO*^{IEC} mice. (B) Western blot analysis of intestinal tissue lysates from WT, *Ufbp1*$^{\Delta/\Delta IEC}$, *IRE1α*$^{\Delta/\Delta IEC}$, and *DKO*^{IEC} mice. Blots shown were representative of two biological replicate samples. GAPDH was used as the loading control. (C) Quantitation of Sec24D protein level in the small intestine tissues. Data are presented as mean ± SEM (n = 3). Statistical significance was determined using one-way ANOVA with Tukey's post hoc test. $^{**}P < 0.01$; $^{***}P < 0.001$ (n = 4).

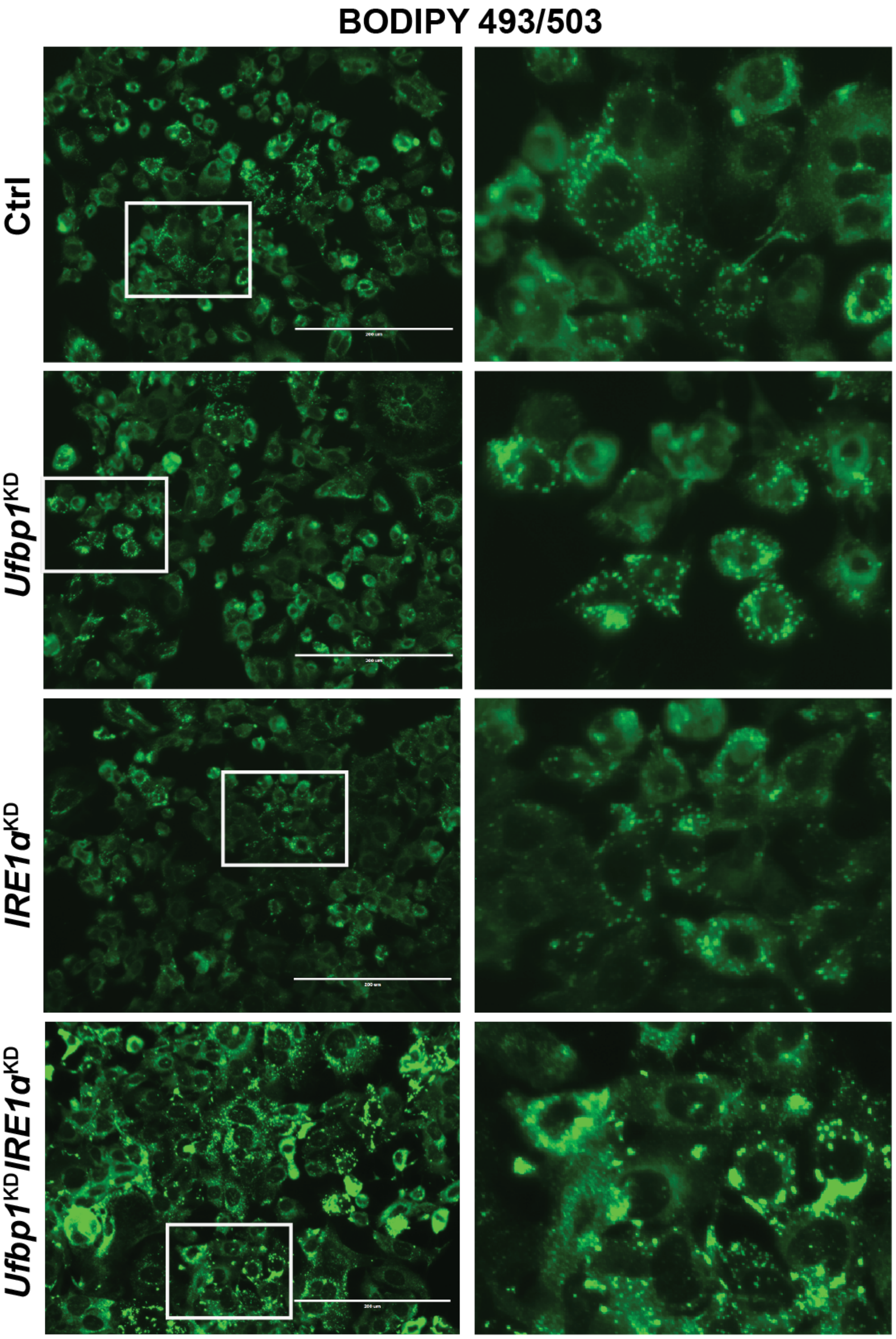
BODIPY 493/503
Ctrl
Ufbp1KD
IRE1αKD
Ufbp1KDIRE1αKD

Figure S5. Representative fluorescence microscopy images of control, *Ufbp1*^{KD}, *IRE1*α^{KD}, and *Ufbp1*^{KD}*IRE1*α^{KD} C2BBe1 cells that were treated with 0.5 mM OA for 8 hours and then stained with 0.1 μM BODIPY 493/503 for 15 minutes. Scale bar, 200 μm.

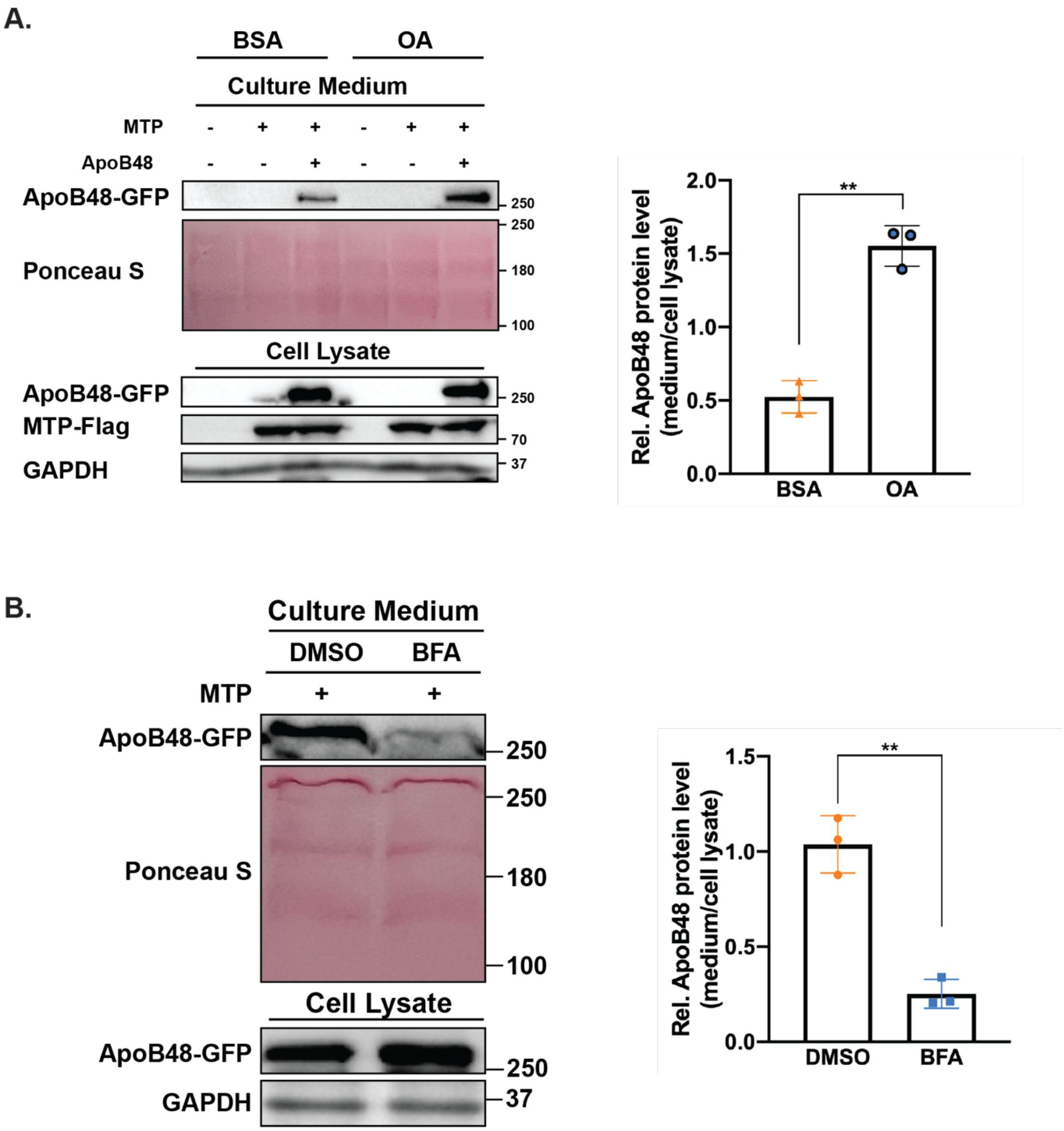
A.
BSA
OA
Culture Medium
MTP
ApoB48
ApoB48-GFP
Ponceau S
250
180
100
Cell Lysate
ApoB48-GFP
MTP-Flag
GAPDH
250
70
37
Rel. ApoB48 protein level (medium/cell lysate)
2.0
1.5
1.0
0.5
0.0
**
BSA
OA
B.
Culture Medium
DMSO
BFA
MTP
ApoB48-GFP
Ponceau S
250
180
100
Cell Lysate
ApoB48-GFP
GAPDH
37
Rel. ApoB48 protein level (medium/cell lysate)
1.5
1.0
0.5
0.0
**
DMSO
BFA

Figure S6. (A) Basal and OA-induced ApoB48-GFP secretion in COS7 cells. ApoB48-GFP plasmid were transfected into COS7 cells stably expressing MTP-Flag protein. After 36h, cells were incubated with the medium containing either BSA or 0.5 mM OA complexed with BSA. Media were collected after an overnight incubation. Both media and cell lysates were subjected to immunoblotting analyses. The images were acquired by Bio-Rad ChemiDoc MP and quantitated with ImageLab software (Bio-Rad). Ponceau S and GAPDH served as loading controls. Data are presented as mean ± SEM (n = 3) from three independent experiments. Statistical significance was determined using Independent-Samples *t* test. $^{**}P < 0.0101$. (B) ApoB48-GFP secretion was blocked by Brefeldin A (BFA). ApoB48-GFP plasmid were transfected into COS7 cells stably expressing MTP-Flag. After 36 hours, cells were pretreated with DMSO or BFA (1 μM for 2 h, followed by incubation with 0.5 mM OA for an additional 8 h before collecting the culture medium and cell lysates. Ponceau S and GAPDH served as loading controls. Data were presented as mean ± SEM (n = 3) from three independent experiments. Statistical significance was determined using Independent-Samples *t* test. $^{**}p < 0.01$.

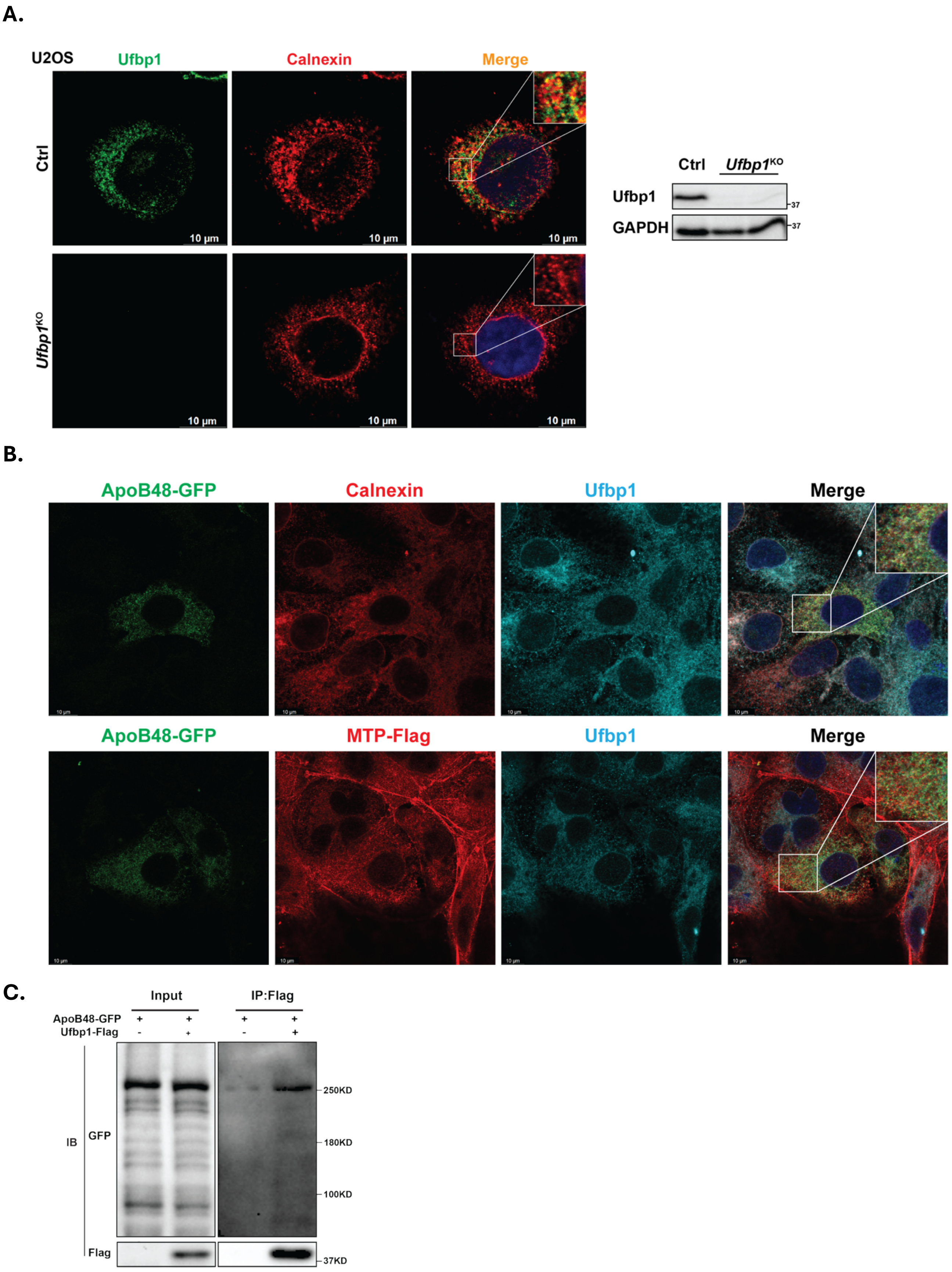
A.
U2OS
Ufbp1
Calnexin
Merge
Ctrl
Ufbp1KO
10 μm
Ctrl
Ufbp1KO
Ufbp1
GAPDH
37
37
B.
ApoB48-GFP
Calnexin
Ufbp1
Merge
ApoB48-GFP
MTP-Flag
Ufbp1
Merge
C.
Input
IP:Flag
ApoB48-GFP
Ufbp1-Flag
IB
GFP
Flag
250KD
180KD
100KD
37KD

Figure S7. (A) Validation of Ufbp1 antibody specificity. WT and Ufbp1 KO U2OS cells were subjected to immunostaining of Ufbp1 and Calnexin (ER marker). Confocal images were acquired by Leica Stellaris 5 confocal microscope with 63x lens and analyzed with Leica LAX S software (Leica Microsystems, Inc., Bannockburn, IL, USA). Western blotting confirmed knockout of Ufbp1 protein. Lack of Ufbp1 signal in Ufbp1 KO cells confirmed the Ufbp1 antibody specificity. (B) Co-localization of ApoB48-GFP and Ufbp1 with Calnexin or MTP-Flag. COS7 cells stably expressing MTP-Flag were plated on glass coverslips overnight and then transiently transfected with ApoB48-GFP plasmid. After 24-hour incubation, cells were immunostained with indicated antibodies. (C) Co-IP assay of Ufbp1 and ApoB-GFP proteins. HEK293T cells were transiently co-transfected with ApoB48-GFP and Flag-Ufbp1. Cell lysates were immunoprecipitated with anti-Flag antibody and analyzed by immunoblotting using the indicated antibodies.

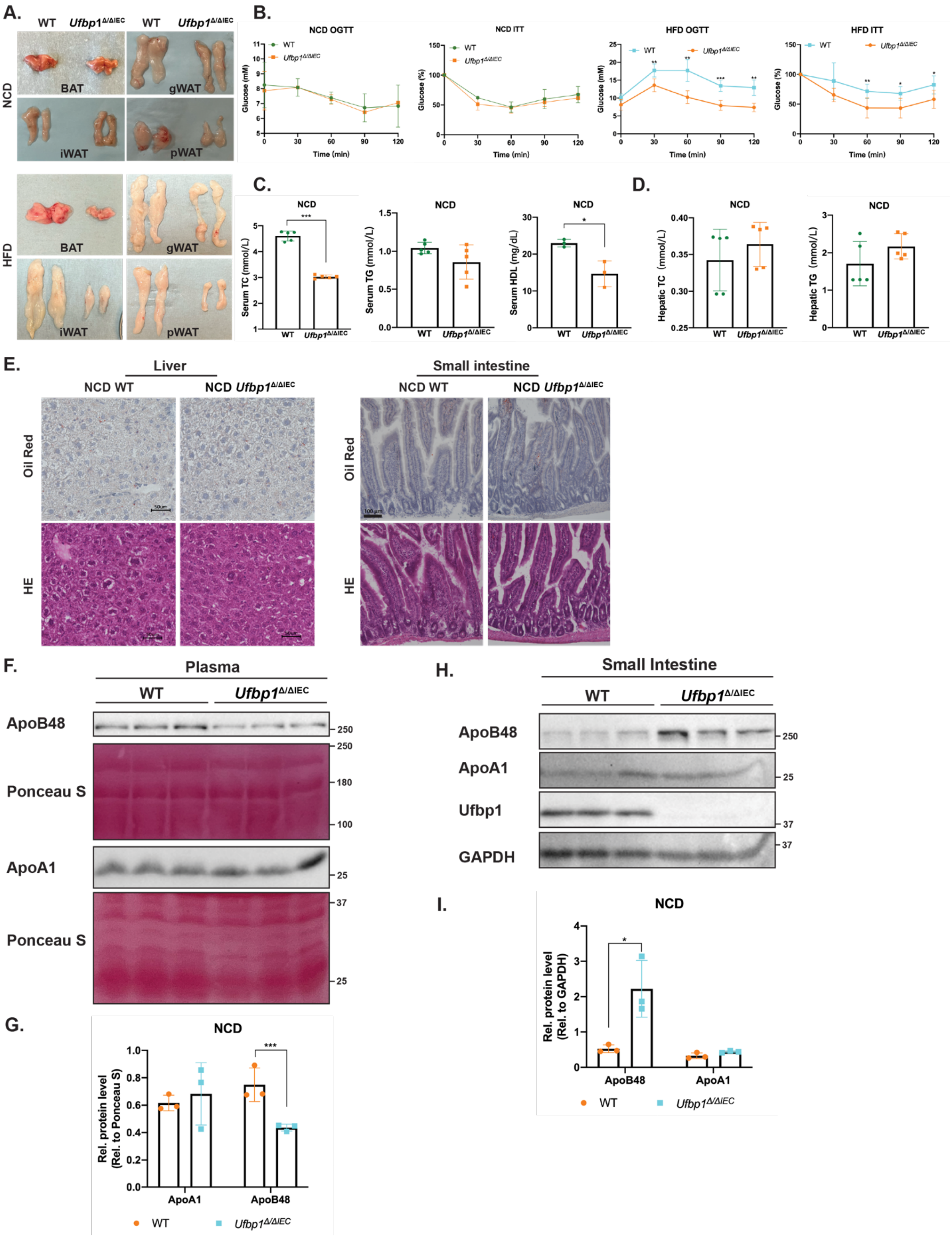

A.
WT
Ufbp1Δ/ΔIEC
NCD
HFD
BAT
gWAT
iWAT
pWAT
B.
NCD OGTT
NCD ITT
HFD OGTT
HFD ITT
Glucose (mM)
Glucose (%)
Time (min)
C.
NCD
Serum TC (mmol/L)
Serum TG (mmol/L)
Serum HDL (mg/dL)
D.
Hepatic TC (mmol/L)
Hepatic TG (mmol/L)
E.
Liver
Small intestine
NCD WT
NCD Ufbp1Δ/ΔIEC
Oil Red
HE
F.
Plasma
ApoB48
Ponceau S
ApoA1
G.
Rel. protein level (Rel. to Ponceau S)
H.
Small Intestine
Ufbp1
GAPDH
I.
Rel. protein level (Rel. to GAPDH)

Figure S8. (A) Representative images of intra-abdominal fat and isolated fat pads from male WT and *Ufbp1*$^{\Delta/\Delta IEC}$ mice on NCD or HFD for 13 weeks. BAT: brown adipose tissue; iWAT: inguinal white adipose tissue; gWAT: gonadal white adipose tissue; pWAT: perirenal white adipose tissue. (B) Oral glucose tolerance test (OGTT) and insulin tolerance test (ITT) of male WT or *Ufbp1*$^{\Delta/\Delta IEC}$ mice on NCD or HFD for 13 weeks. Statistical significance was determined using Independent-Samples *t* test. **P* < 0.05; ***P* < 0.01; ****P* < 0.001 (n = 5). (C) Plasma TC, TG, and HDL levels in NCD-fed mice (n = 5). (D) Hepatic TC and TG of male WT or *Ufbp1*$^{\Delta/\Delta IEC}$ mice on NCD for 13 weeks (n = 5 mice per group). Statistical significance was determined using Independent-Samples *t* test. **P* < 0.05; ***P* < 0.01; ****P* < 0.001. (E) Oil-Red-O and H&E staining of liver and small intestine tissues of male WT or *Ufbp1*$^{\Delta/\Delta IEC}$ on NCD for 13 weeks. Scale bar, 50 μm. (F and H) Immunoblotting of ApoB48 and ApoA1 proteins in the plasma and small intestine tissues of NCD-fed WT and *Ufbp1*$^{\Delta/\Delta IEC}$ mice. (G and I) Quantitation of relative levels of ApoB48 and ApoA1 in the plasma and small intestine tissues. Data are represented as mean ± SEM. Statistical significance was determined using Independent-Samples *t* test. ****P* < 0.001 (n = 3).

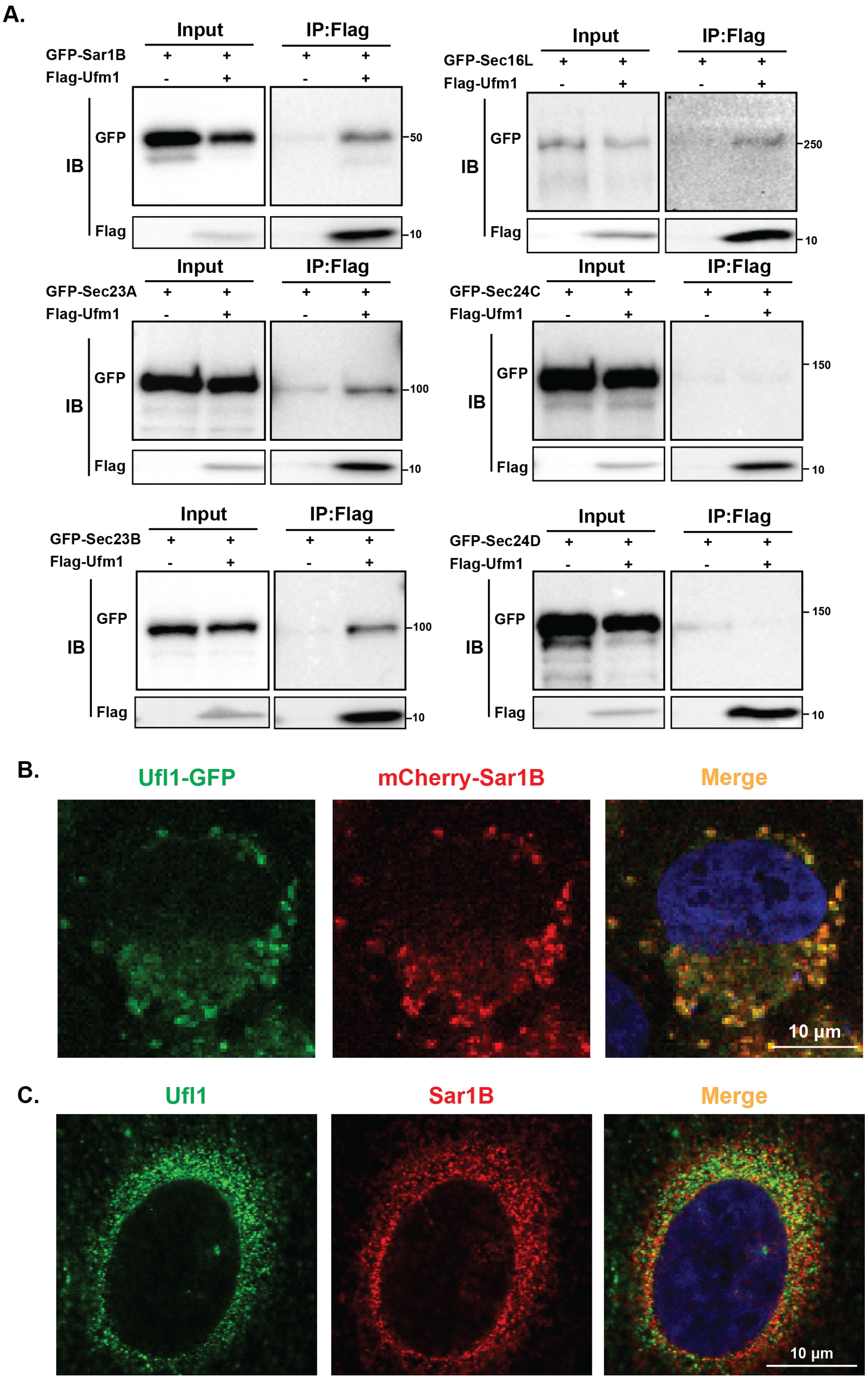
A.
Input
IP:Flag
GFP-Sar1B
Flag-Ufm1
IB
GFP
Flag
50
10
GFP-Sec16L
250
GFP-Sec23A
100
GFP-Sec24C
150
GFP-Sec23B
GFP-Sec24D
B.
Ufl1-GFP
mCherry-Sar1B
Merge
10 μm
C.
Ufl1
Sar1B

Figure S9. (A) Co-IP assays of Flag-Ufm1 and COPII components. HEK293T cells were transiently co-transfected with Flag-Ufm1, and GFP-Sar1, GFP-Sec23A, GFP-Sec23B, GFP-Sec16L, GFP-Sec24C and GFP-Sec24D respectively. Cell lysates were immunoprecipitated with anti-Flag antibody and analyzed by immunoblotting using the indicated antibodies. (B) Co-localization of mCherry-Sar1B and Ufl1-GFP proteins in U2OS cells. U2OS cells were transfected with mCherry-Sar1B and Ufl1-GFP. Images were captured using a Leica Stellaris 5 confocal microscope. Scale bar, 10 μm. (C) Co-localization of endogenous Sar1B and Ufl1 proteins in U2OS cells. U2OS cells were subjected to immunofluorescence staining of Sar1B and Ufl1 antibodies. Images were captured using a Leica Stellaris 5 confocal microscope. Scale bar, 10 μm.

Key Resources:

Table S1 Primer for quantitative RT-PCR

Mouse GAPDH	F: CTCCCACTCTTCCACCTTCG
	R: GCCTCTCTTGCTCAGTGTCC
Mouse Sec24D	F: CACCACCGAGTGTGTGATTC
	R: TGTAGGCTTTGCACCTGTTG
Mouse ApoB48	F: CAAGCACCTCCGAAAGTACG
	R: AAGGGTACACTGGTTGGTCC
Human GAPDH	F: AGAAGGCTGGGGCTCATTTG
	R: AGGGGCCATCCACAGTCTTC
Human Sec24D	F: CACCTGTCAACAATGTGGCA
	R: GCAAAATGGAAGGCTGTGGA

Table S2 gRNA and shNA sequences

Human Ufbp1 gRNA	GTAGCGGCGGCTCTGCTAGT
Human IRE1α gRNA	CTTGTTGTTTGTGTCAACGC
Human Ufm1 gRNA#1	TCACGCTGACGTCGGACCCA
Human Ufm1 gRNA#2	CTTTAAGATCACGCTGACGT
Human Uba5 gRNA#1	TCCCGAGGAGCGGCGACGGA
Human Uba5 gRNA#2	GCTGGAGCGGGAACTTGCCC
Human Sec24D shRNA#1	GTAGCGGCGGCTCTGCTAGT
Human Sec24D shRNA#2	CTTGTTGTTTGTGTCAACGC

Table S3 Antibodies Detail

Antibody	Source	Cat #	RRID
ApoA1	ProteinTech	14427-1-AP	AB_2056524
ApoB	ProteinTech	20578-1-AP	AB_10732938
Flag	Sigma	F4049	AB_439701
GAPDH	Santa Cruz Biotech	sc-25778	AB_10167668
GFP	Cell Signaling	2555	AB_10692764

IRE1α	Cell Signaling	3294	AB_823545
PLIN2	Santa Cruz Biotech	sc-377429	AB_3661746
SEC24D	Cell Signaling	14687	AB_2798574
Sar1B	ProteinTech	22292-1-AP	AB_11182713
Ufbp1	ProteinTech	15783-1-AP	AB_2827383
Ufm1	Abcam	ab109305	AB_10864675
Uba5	Li Lab	N/A	N/A
Ufl1	Li Lab	N/A	N/A
Ufc1	ProteinTech	15783-1-AP	AB_2213938
β-actin	Li Lab	N/A	N/A
c-Myc (9E10)	Santa Cruz Biotech	sc-40	AB_627268
Goat anti-Rabbit IgG, HRP-	Jackson ImmunoResearch	111-035-144	AB_2307391
Goat anti-Mouse IgG, HRP-	Jackson ImmunoResearch	115-035-146	AB_2307392
Goat anti-Rabbit IgG, AF488-	Jackson ImmunoResearch	111-545-144	AB_2338052
Goat anti-Mouse IgG, AF488-	Jackson ImmunoResearch	115-545-166	AB_2338852
Goat anti-Rabbit IgG, Cy3-	Jackson ImmunoResearch	111-165-144	AB_2338006
Goat anti-Mouse IgG, Cy3-	Jackson ImmunoResearch	115-165-166	AB_2338692

Table S4 Bacterial and virus strains

Escherichia coli XL-1 Blue

Table S5 Chemicals, Peptides, and Recombinant Proteins

Reagent	Source	Cat #
Brefeldin A (BFA)	Cayman Chemical	Cat#11861
BODIPY 493/503	Cayman Chemical	Cat# 25892

Bovine Serum Albumin (BSA) Fatty acid-free powder	Thermo Fisher	Cat#4693116001
Bovine Serum Albumin (BSA) (Fraction V)	Thermo Fisher	Cat# BP1600-100
DMEM/High Glucose	Hyclone	Cat# SH30022.01
Fetal Bovine Serum (FBS)	Hyclone	Cat# Sh30396.03
Glucose	Thermo Fisher	Cat# 15023021
Insulin	Thermo Fisher	Cat# 12585014
Oleic acid	Sigma-Aldrich	Cat# O1383
Oil Red	Sigma-Aldrich	Cat# O0625
Opti-MEM™ I	Gibco	Cat#31985088
PEI	Polysciences, Inc.	Cat#23966-1
Penicillin-Streptomycin	Hyclone	Cat# SV30010
Ponceau S	ThermoFisher	Cat# 6226-79-5
Protease inhibitor cocktail	Roche	Cat# HY-K0010
Puromycin	MedChemExpress	Cat# HY-B1743
0.25 % Trypsin	Corning	Cat# 25-053-CI

Table S6 DNA, RNA prep and assay kits

Kit	Source	Cat #
E.Z.N.A.® HP Total RNA Kit	Omega Bio-tek	Cat# R6812-02
E.Z.N.A.® Plasmid DNA Mini Kit I	Omega Bio-tek	Cat# D6942-02
High-capacity cDNA RT kit	ThermoFisher	Cat# 4368814
2x Green qPCR Master Mix	APExBIO	Cat# K1070
Glucose Monitor Kit	Metene	Cat# TD-4116
Lipid Panel	Piccolo Express Chemistry Analyzer	Cat# 400-1025
Micro BCA Protein Assay Kits	Thermo Fisher	Cat# 23232

Triglyceride Assay Kit	Novus Biologicals	Cat# NBP3-24540
Total Cholesterol Assay Kit	Novus Biologicals	Cat# NBP3-25838

Table S7 Experimental Models: Cell Lines

Human: HEK293T cells	ATCC	Cat# CRL-1573
Human: C2Bbe1 cells	ATCC	Cat# CRL-2102
Human: U2OS cells	ATCC	Cat# HTB-96
Cercopithecus aethiops: COS7	ATCC	Cat# CRL-1651

Table S8 Mouse strains

Mouse: Ufbp1$^{f/f}$: B6.	Li Lab	
Mouse: IRE1$\alpha^{f/f}$: B6.	Dr. Randal Kauffman's lab	
Mouse: PERK$^{f/f}$: B6.	Jackson Laboratory	# 023006
Mouse: Villin-Cre: B6.	Dr. Sylvie Robine's lab	

Table S9 Plasmids and Recombinant DNA

pRP.ExTri-CMV-5'UTR-ApoB48-Linker-eGFP	Addgene	Cat# 138334
hMTP-Flag	Addgene	Cat# 138335
pCDH-hMTP-Flag	This paper	N/A
lentiCRISPR Ufm1 gRNA	This paper	N/A
lentiCRISPR Uba5 gRNA	This paper	N/A
lentiCRISPR Ufbp1 gRNA	This paper	N/A
lentiCRISPR IRE1α gRNA	This paper	N/A
pLKO.1-Sec24D shRNA#1	This paper	N/A
pLKO.1-Sec24D shRNA#2	This paper	N/A
PCMV-hUfm1-Myc	This paper	N/A

PCMV-hUba5-Myc	This paper	N/A
PCMV-hUfc1-Myc	This paper	N/A
PCMV-hUfl1-Myc	This paper	N/A
PCMV-hUfbp1-Flag	This paper	N/A
PCDH-hSar1-Flag	This paper	N/A
PCDH-hSar1-GFP	This paper	N/A
PCDH-hSec23A-GFP	This paper	N/A
PCDH-hSec23B-GFP	This paper	N/A
PCDH-hSec23B-Flag	This paper	N/A
PCDH-hSec24C-GFP	This paper	N/A
PCDH-hSec24D-GFP	This paper	N/A
PCDH-hSec16L-GFP	This paper	N/A

Table S10 Softwares and Algorithms

Adobe Illustrator 2025	Adobe	https://www.adobe.com/product/photoshop.html
Adobe Photoshop CC 2025	Adobe	https://www.adobe.com/product/photoshop.html
GraphPad Prism 9	GraphPad Software	https://www.graphpad.com
IBM SPSS Statistics 22	IBM, Chicago, IL, USA	https://www.ibm.com/analytics/us/en/technology/spss/
ImageJ	NIH, USA	https://imagej.nih.gov/ij/
OriginLab 2025	OriginLab	https://www.originlab.com
